# Association of Prior Intracerebral Hemorrhage With Major Adverse Cardiovascular Events

**DOI:** 10.1001/jamanetworkopen.2022.34215

**Published:** 2022-10-03

**Authors:** David Gaist, Stine Munk Hald, Luis Alberto García Rodríguez, Anne Clausen, Sören Möller, Jesper Hallas, Rustam Al-Shahi Salman

**Affiliations:** 1 Research Unit for Neurology, Odense University Hospital, Odense, Denmark; University of Southern Denmark, Odense, Denmark; 2 Centro Español Investigación Farmacoepidemiológica, Madrid, Spain; 3 Open Patient Data Explorative Network, Odense University Hospital, Odense, Denmark; 4 Department of Clinical Research, University of Southern Denmark, Odense, Denmark; 5 Department of Clinical Pharmacology, Pharmacy and Environmental Medicine, University of Southern Denmark, Odense, Denmark; 6 Centre for Clinical Brain Sciences, University of Edinburgh, Edinburgh, United Kingdom

## Abstract

**Importance:**

Patients with stroke due to nontraumatic (spontaneous) intracerebral hemorrhage (ICH) often harbor vascular risk factors and comorbidities, but it is unclear which major adverse cardiovascular events (MACEs) occur more frequently among patients with a prior ICH than the general population.

**Objective:**

To evaluate the risk of a MACE for patients with a prior ICH compared with the general population.

**Design, Setting, and Participants:**

This cohort study identified 8991 patients with a first ICH in the Danish Stroke Registry from January 1, 2005, to June 30, 2018, who were aged 45 years or older and survived more than 30 days after an ICH. Patients in this ICH cohort were matched 1:40 on age, sex, and ICH-onset date with a comparison cohort of 359 185 individuals from the general population without a prior ICH. Both cohorts were followed up for 6 months or more until December 31, 2018, for outcomes using registry data. Data were analyzed from October 1, 2021, to July 19, 2022.

**Exposures:**

Intracerebral hemorrhage identified by a nationwide clinical database.

**Main Outcomes and Measures:**

The main outcomes were ICH, ischemic stroke, myocardial infarction, and a composite of MACEs. For each outcome, a case-control study nested within the cohorts was also performed, adjusting for time-varying exposures and potential confounders. Crude absolute event rates per 100 person-years, adjusted hazard ratios (aHRs) and 95% CIs and, in the nested case-control analyses, crude and adjusted odds ratios and 95% CIs were calculated.

**Results:**

The ICH cohort (n = 8991; 4814 men [53.5%]; mean [SD] age, 70.7 [11.5] years) had higher event rates than the comparison cohort (n = 359 185; 192 256 men [53.5%]; mean [SD] age, 70.7 [11.5] years) for MACEs (4.16 [95% CI, 3.96-4.37] per 100 person-years vs 1.35 [95% CI, 1.33-1.36] per 100 person-years; aHR, 3.13 [95% CI, 2.97-3.30]), ischemic stroke (1.52 [95% CI, 1.40-1.65] per 100 person-years vs 0.56 [95% CI, 0.55-0.57] per 100 person-years; aHR, 2.64 [95% CI, 2.43-2.88]), and ICH (1.44 [95% CI, 1.32-1.56] per 100 person-years vs 0.06 [95% CI, 0.06-0.07] per 100 person-years; aHR, 23.49 [95% CI, 21.12-26.13]) but not myocardial infarction (0.52 [95% CI, 0.45-0.60] per 100 person-years vs 0.48 [95% CI, 0.47-0.49] per 100 person-years; aHR, 1.12 [95% CI, 0.97-1.29]). Nested case-control analyses returned risk estimates of similar magnitude as the cohort analyses.

**Conclusions and Relevance:**

The findings of this cohort study suggest that Danish patients with a prior ICH had statistically significantly higher rates of MACEs than the general population, indicating a need for attention to optimal secondary prevention with blood pressure lowering and antithrombotic and statin therapies after an ICH in clinical research and practice.

## Key Points

**Question:**

What is the risk of major adverse cardiovascular events (MACEs) among patients with a prior intracerebral hemorrhage compared with the general population?

**Findings:**

In this cohort study, 8991 patients with a prior intracerebral hemorrhage had statistically significantly higher rates of MACEs, ischemic stroke, and intracerebral hemorrhage, but not myocardial infarction, than 359 185 age- and sex-matched general population controls.

**Meaning:**

The results of this study suggest that there is a need for effective secondary vascular prevention after intracerebral hemorrhage.

## Teaser

This cohort study evaluates the risk of major adverse cardiovascular events for patients in Denmark with a prior intracerebral hemorrhage (ICH) compared with the general population.

## Introduction

Studies of patients with an intracerebral hemorrhage (ICH) have documented future risks of recurrent ICH, ischemic stroke (IS), and myocardial infarction (MI) among these patients.^1,2,3,4^ Fewer studies have compared the risk of arterial ischemic events among patients with a prior ICH with the risk of arterial ischemic events among people without an ICH.^5,6,7^ A recent study from the US based on pooled data from 4 population-based cohorts reported that, during long-term follow-up, IS was approximately 3 times as common and MI twice as common among patients with a prior ICH, compared with participants without an ICH.^6^ Another recent study reported a similar increase in the risk of IS after an ICH in a cohort in the United Kingdom.^7^ The findings of these studies^6,7^ reemphasize the need for better prevention of ischemic events after an ICH.^8^ However, the ICH cohorts in these studies were relatively small (318 in the US study^6^ and 988 in the UK study^7^), data on the risk of an ICH during follow-up were limited^6^ (or not provided^7^), and the studies did not describe other major adverse cardiovascular events (MACEs). Furthermore, to our knowledge, there are few data concerning the severities of these outcomes after an ICH,^9^ which is a relevant consideration in addition to their frequency, when considering the risks and benefits associated with secondary prevention agents.

Therefore, we aimed to assess the risk of MACEs in a large number of patients with a prior ICH to investigate absolute and relative risks of all ischemic and hemorrhagic MACEs, improve the precision of these estimates, adjust for important confounders, explore associations between patients’ comorbidities (eg, atrial fibrillation [AF], diabetes, and hypertension) and these outcomes, and compare the severities of recurrent ICH and IS after an ICH.

## Methods

We used a long-standing, nationwide, prospectively collected stroke registry in Denmark^10^ to identify a cohort of patients with first-ever ICH to be matched with a general population comparison cohort (eMethods and eFigure 1 in the Supplement). We followed up both cohorts for outcomes using nationwide Danish registries and calculated absolute and relative outcome rates. For each primary outcome, we also conducted a nested case-control analysis, calculating relative rates while adjusting for time-varying covariates (such as comorbidities and antithrombotic drug use). In accordance with Danish law regarding register-based research, the study was approved by the Region of Southern Denmark, and informed consent was waived^11^ because data were pseudonymized. This study followed the Strengthening the Reporting of Observational Studies in Epidemiology (STROBE) reporting guideline.

### Setting and Data Sources

In Denmark (population 5.8 million), health services are tax funded and free of charge for all residents, who have a unique civil registration number allowing their unambiguous linkage to nationwide administrative and medical registries.^12^ We retrieved data from 4 sources: the Danish Stroke Registry (Stroke Registry; operational since 2003), the Danish National Patient Registry^13^ (Patient Registry), the Danish National Prescription Registry (Prescription Registry),^14^ and the Civil Registration System.^15^ The Civil Registration System holds data on the civil registration number, migration history, and vital status (including date of death) of all residents of Denmark.

In Denmark, it is compulsory for departments evaluating patients within 7 days of stroke onset to report standardized data to the Stroke Registry. The Stroke Registry records symptomatic strokes.^16,17^ We sought patients with spontaneous ICH (ie, not due to trauma, underlying malignant neoplasm, venous thrombosis, or vascular malformations), using codes that have a high positive predictive value (PPV) for spontaneous ICH (82%) in the Stroke Registry,^16^ which also contains information on severity of stroke on admission according to the Scandinavian Stroke Scale (SSS) score.^18^

### ICH Cohort and Comparison Cohort

The source population was all people aged 45 years or older in Denmark from January 1, 2005, to December 31, 2018. The ICH cohort comprised patients from the source population recorded in the Stroke Registry with admissions for a first-ever ICH from January 1, 2005, through June 30, 2018. Because of the high early case fatality rate after and ICH,^19^ when coding of readmissions is less accurate in the Stroke Registry,^20^ we included only patients who survived to day 31 after ICH onset, after which follow-up began (henceforth referred to as the *start date*). We matched each patient in the ICH cohort on sex, birth year, and start date with 40 individuals from the general population (comparison cohort) via use of the Civil Registration System^15^ (eFigure 1 in the Supplement). We excluded people from the cohorts if, at start of follow-up, they had a diagnosis of ICH in the Stroke Registry before the event qualifying for inclusion in the ICH cohort or they had been residents of Denmark for less than 10 years (to account for all relevant medical history, including use of medications).

### Follow-up

Follow-up began on the start date and ended on the date of an outcome, emigration, death, or end of the follow-up period (December 31, 2018), whichever came first. We retrieved date of death (or emigration) from the Civil Registration System.^15^

The date of an outcome was the first occurrence during follow-up of an individual event (IS, ICH, or MI) or the first occurrence of any individual component of the composite MACE outcome. We also quantified the frequency of multiple outcomes during follow-up (see Supplementary Cohort Analyses below).

### Outcomes

The 4 primary outcomes were IS, ICH, MI, and MACE (defined as stroke [IS, ICH, or unspecified stroke], nonfatal MI, systemic embolism, or vascular death). Vascular death was defined as death within 30 days of a hospital discharge during follow-up for 1 or more of the following: stroke (ie, ICH, IS, or unspecified stroke) or other cardiovascular events (ie, intracranial extra-axial hemorrhage [subdural hematoma, subarachnoid hemorrhage, epidural hematoma, or intracranial hemorrhage unspecified]; MI; systemic embolism; revascularization procedures; mesenteric ischemia; sudden cardiac death; venous thromboembolism [deep vein thrombosis or pulmonary embolism]; or extracranial hemorrhage [defined as gastrointestinal bleeding (upper, lower, or unspecified), acute bleeding anemia, hematuria, hemopericardium, esophageal varicose vein hemorrhage, esophageal hemorrhage, peritoneal hemorrhage, hemothorax, hemorrhage in bile duct or pancreas, or hemorrhage in spinal cord]).

The 13 secondary outcomes were the following individual and composite types of vascular events: stroke (IS, ICH, or unspecified stroke); intracranial extra-axial hemorrhage; systemic embolism; mesenteric ischemia; venous thromboembolism; extracranial hemorrhage; sudden cardiac death; major ischemic vascular event (IS, MI, unspecified stroke, systemic embolism, revascularization procedures, mesenteric ischemia, venous thromboembolism, or sudden cardiac death); major hemorrhagic vascular event (intracranial extra-axial hemorrhage and extracranial hemorrhage); major arterial event (IS, MI, unspecified stroke, systemic embolism, revascularization procedures, mesenteric ischemia, or sudden cardiac death); death from any cause; vascular death; and nonvascular death (death not classified as vascular death).

We ascertained strokes through the Stroke Registry and all other events through the Patient Registry^13^ (codes in eTable 1 in the Supplement). To ensure high PPV, we considered only hospital admissions with primary diagnosis codes as events.^13^

### Covariates

We used information from the Patient Registry and the Prescription Registry for a fixed 10-year period preceding the date of ICH onset (and the same dates in the matched comparison cohort) (eFigure 2 in the Supplement) to classify the following potential confounders and drug exposures before the start of follow-up (code lists in eTable 1 in the Supplement): hypertension, AF, IS, MI, systemic embolism, peripheral arterial disease, venous thromboembolism, diabetes, chronic kidney failure, chronic hepatic diseases, congestive heart failure, cancer, chronic obstructive pulmonary disease, disorders indicative of high alcohol use (henceforth referred to as *high alcohol intake*), and use of medications (separate covariates for each of the following drugs and drug classes: low-dose aspirin, clopidogrel, dipyridamole, direct oral anticoagulants, vitamin K antagonists, statins, thiazides and other nonloop diuretics, loop diuretics, β-blockers, calcium channel blockers, angiotensin-converting enzyme inhibitors and angiotensin-receptor blockers, nonsteroidal anti-inflammatory drugs, selective serotonin reuptake inhibitors, and proton pump inhibitors) (for classification of recency of use of these medications, see eMethods in the Supplement).

### Nested Case-Control Analysis

For each of the primary outcomes (IS, ICH, MI, and MACE), we also conducted a nested case-control analysis. For each outcome (case), we identified up to 40 controls (without replacement) among eligible cohort members, matched on age and sex using risk-set sampling. Controls were allotted the date of the outcome (index date) of their respective case. In accordance with standard practice, we allowed that cases could be sampled as controls until their case-defining event. We classified the same potential confounders and exposures to medications as listed under Covariates using all information available up to the index date (eFigure 3 in the Supplement). The nested case-control analysis allowed us to incorporate time-dependent covariates—such as current use of a variety of drugs—without the unduly complex exposure handling of a corresponding cohort study.

### Statistical Analysis

#### Cohort Analyses

Characteristics of the study cohorts were summarized using descriptive statistics. We derived figures for the cumulative incidence in the ICH cohort and comparison cohort for each of the outcomes based on Kaplan-Meier analyses of the available follow-up; also, we derived figures that accounted for death as competing event (Aalen Johansen estimator). For stroke outcomes (IS, ICH, and unspecified stroke), we compared stroke severity (classified based on the SSS score at the time of admission as recorded in the Stroke Registry) and case fatality (1 day, 7 days, and 30 days) across outcomes using the χ^2^ test.

We calculated the absolute rates (incidence rates [IRs]; ie, number of events divided by person-years at risk) and corresponding 95% CIs for each outcome within each cohort, overall and within subgroups defined by baseline risk factors (sex, age [45-59, 60-74, 75-84, and ≥85 years], history of AF, IS, MI, diabetes, chronic kidney failure, chronic hepatic diseases, chronic obstructive pulmonary disease, and high alcohol intake). We also calculated annual IRs for each of the first 5 years of follow-up. Corresponding 95% CIs were calculated under the assumption of a Poisson distribution.

We used Cox proportional hazards regression models to calculate the HR and corresponding 95% CIs for each outcome in patients in the ICH cohort with reference to people in the comparison cohort, overall and within the subgroups listed. We calculated unadjusted hazard ratios (HRs) and adjusted HRs (aHRs) for sex, age (5-year bands), study period (5-year bands), and the potential confounders and exposures listed under Covariates. For main outcomes, we also performed competing risk analyses with death as the competing event to calculate Fine-Gray subdistribution HRs. Finally, as a simple metric for the potential association of unmeasured confounding, we calculated the E-value.^21^ The E-value is the minimum strength of association that an unmeasured confounder would need to have with both the exposure and the outcome to fully explain the observed association. With higher values, the observed association is less likely to be explained by unmeasured confounding.

#### Supplementary Cohort Analyses

In the main analysis, we performed a single follow-up for IS, ICH, and MI, whichever event came first. We also performed a sensitivity analysis in which we had followed up separately for each outcome (eg, IS oblivious to all other outcome events) and calculated the resulting number of events and event rates to compare them with corresponding data from the main analyses.

#### Nested Case-Control Analyses

We used conditional logistic regression to calculate odds ratios and 95% CIs for the risk of each of the outcomes (ie, separate models for each outcome), adjusted for covariates (as listed under Covariates), assessed based on all available prior information up to the index date (date of outcome for cases and same date for their corresponding controls) (eFigure 3 in the Supplement). The nested case-control analyses are further detailed in the eMethods in the Supplement.

A 2-tailed *P* < .05 was considered statistically significant. All analyses were performed using Stata SE software, version 17.0 (StataCorp LLC) and the E-value commands in Stata. Data were analyzed from October 1, 2021, to July 19, 2022.

## Results

We included 8991 patients in the ICH cohort (4814 men [53.5%]; mean [SD] age, 70.7 [11.5] years) and 359 185 age- and sex-matched individuals in the comparison cohort (192 256 men [53.5%]; mean [SD] age, 70.7 [11.5] years) (Table 1). The baseline prevalence of all disorders included in the present analyses (apart from history of cancer) was higher in the ICH cohort than in the comparison cohort, notably for hypertension (6084 [67.7%] vs 185 103 [51.5%]), previous IS (1219 [13.6%] vs 11 362 [3.2%]), AF (1324 [14.7%] vs 27 392 [7.6%]), and high alcohol intake (839 [9.3%] vs 12 235 [3.4%]) (Table 1). Use of antiplatelet agents (2565 [28.5%] vs 72 407 [20.2%]) and oral anticoagulants (998 [11.1%] vs 17 546 [4.9%]) was also more frequent in the ICH cohort than in the comparison cohort.

**Table 1.** Baseline Characteristics of Study Cohorts

Characteristic	Cohort, No. (%)	
	ICH (n = 8991)	Comparison (n = 359 185)
Sex		
Male	4814 (53.5)	192 256 (53.5)
Female	4177 (46.5)	166 929 (46.5)
Age, mean (SD), y	70.7 (11.5)	70.7 (11.5)
Age group, y		
45-59	1827 (20.3)	72 711 (20.2)
60-74	3689 (41.0)	147 903 (41.2)
75-84	2500 (27.8)	99 488 (27.7)
≥85	975 (10.8)	39 083 (10.9)
Calendar year		
2005-2011	4849 (53.9)	193 657 (53.9)
2012-2018	4142 (46.1)	165 528 (46.1)
Comorbidity at baseline^a^		
Hypertension	6084 (67.7)	185 103 (51.5)
Atrial fibrillation	1324 (14.7)	27 392 (7.6)
Previous ischemic stroke	1219 (13.6)	11 362 (3.2)
Previous myocardial infarction	402 (4.5)	13 849 (3.9)
Systemic embolism	33 (0.4)	884 (0.2)
Peripheral arterial disease	350 (3.9)	10 432 (2.9)
Venous thromboembolism	285 (3.2)	8323 (2.3)
Diabetes	1048 (11.7)	37 233 (10.4)
Chronic kidney failure	192 (2.1)	5235 (1.5)
Chronic hepatic diseases	153 (1.7)	2477 (0.7)
Chronic obstructive pulmonary disorder	1957 (21.8)	75 144 (20.9)
High alcohol intake^b^	839 (9.3)	12 235 (3.4)
Congestive heart failure	456 (5.1)	15 871 (4.4)
Cancer	823 (9.2)	33 490 (9.3)
Medication use at baseline^c^		
Antiplatelet agents	2565 (28.5)	72 407 (20.2)
Low-dose aspirin	2214 (24.6)	66 004 (18.4)
Clopidogrel	441 (4.9)	8741 (2.4)
Other ADP drugs (ticagrelor or prasugrel)	9 (0.1)	387 (0.1)
Oral anticoagulant agents	998 (11.1)	17 546 (4.9)
Vitamin K antagonist	816 (9.1)	13 921 (3.9)
Direct oral antagonist	189 (2.1)	3722 (1.0)
Dabigatran	27 (0.3)	1412 (0.4)
Rivaroxaban	112 (1.2)	1220 (0.3)
Apixaban	50 (0.6)	1094 (0.3)
Edoxaban	<5	26 (0.0)
Statins	2194 (24.4)	78 055 (21.7)
Thiazides and other nonloop diuretics	1308 (14.5)	54 507 (15.2)
Loop diuretics	820 (9.1)	27 825 (7.7)
β-blockers	1704 (19.0)	55 195 (15.4)
Calcium channel blockers	1209 (13.4)	55 606 (15.5)
ACE inhibitors and angiotensin II receptor blockers	2494 (27.7)	94 136 (26.2)
Nonsteroidal anti-inflammatory drugs	740 (8.2)	23 149 (6.4)
Selective serotonin reuptake inhibitors	888 (9.9)	21 035 (5.9)
Proton pump inhibitors	1160 (12.9)	36 596 (10.2)

Abbreviations: ACE, angiotensin-converting enzyme; ADP, adenosine diphosphate; ICH, intracerebral hemorrhage.

^a^ Assessed on date of ICH onset (inclusion date) for ICH cohort and the same date for the matched comparison cohort and based on data in preceding 10 years.

^b^ Diagnoses indicative of high alcohol intake.

^c^ Supply of latest prescription covered the inclusion date or ended less than 30 days before the inclusion date.

### Cohort Analyses

During a total follow-up of 37 482 person-years (mean [SD] follow-up, 4.2 [3.6] years), 571 patients in the ICH cohort had an IS, and 12 416 patients in the comparison cohort had an IS (total follow-up, 2 216 351 person-years; mean [SD] follow-up, 6.2 [3.8] years) (Table 2; eFigure 4 in the Supplement), corresponding to crude IRs per 100 person-years of 1.52 (95% CI, 1.40-1.65) for the ICH cohort and 0.56 (95% CI, 0.55-0.57) for the comparison cohort and an aHR of 2.64 (95% CI, 2.43-2.88). In the ICH cohort, 538 patients had recurrent ICH and in the comparison cohort, 1377 patients had their first-ever ICH during follow-up, corresponding to IRs of 1.44 (95% CI, 1.32-1.56) per 100 person-years for the ICH cohort and 0.06 (95% CI, 0.06-0.07) per 100 person-years for the comparison cohort and an aHR of 23.49 (95% CI, 21.12-26.13). Incidence rates per 100 person-years and aHRs were also higher in the ICH cohort than in the comparison cohort for MACEs (IR, 4.16 [95% CI, 3.96-4.37] vs 1.35 [95% CI, 1.33-1.36]; aHR, 3.13 [95% CI, 2.97-3.30]) but not for MI (IR, 0.52 [95% CI, 0.45-0.60] vs 0.48 [95% CI, 0.47-0.49]; aHR, 1.12 [95% CI, 0.97-1.29]) (Table 2; eFigures 4 and 5 in the Supplement). Risks for all these outcomes (except MI) were higher among patients with or without comorbid AF at baseline (eFigure 6 in the Supplement).

**Table 2.** Absolute and Relative Rates of Main and Secondary Study Outcomes in the Study Cohorts

Outcome during follow-up	Absolute incidence rate				Adjusted incidence rate difference^a^	Relative rate with comparison cohort as reference, aHR (95% CI)^a^	E-value
	ICH cohort		Comparison cohort				
	No. of events/person-years	Event rate per 100 person-years (95% CI)	No. of events/person-years	Event rate per 100 person-years (95% CI)	Event rate per 100 person-years (95% CI)		
Primary outcomes and select secondary outcomes							
Ischemic stroke	571/37 482	1.52 (1.40-1.65)	12 416/2 216 351	0.56 (0.55-0.57)	0.92 (0.80-1.05)	2.64 (2.43-2.88)	4.72
ICH	538/37 482	1.44 (1.32-1.56)	1377/2 216 351	0.06 (0.06-0.07)	1.35 (1.21-1.51)	23.49 (21.12-26.13)	46.49
Unspecified stroke (secondary outcome)	51/37 269	0.14 (0.10-0.18)	755/2 212 963	0.03 (0.03-0.04)	0.07 (0.05-0.11)	3.39 (2.53-4.53)	6.24
Myocardial infarction	194/37 482	0.52 (0.45-0.60)	10 669/2 216 351	0.48 (0.47-0.49)	0.06 (−0.01-0.14)	1.12 (0.97-1.29)	1.49
MACE^b^	1547/37 208	4.16 (3.96-4.37)	29 780/2 210 939	1.35 (1.33-1.36)	2.88 (2.66-3.11)	3.13 (2.97-3.30)	5.71
Vascular death (secondary outcome)^c^	207/37 269	0.56 (0.48-0.64)	4438/2 212 963	0.20 (0.19-0.21)	0.44 (0.36-0.54)	3.20 (2.78-3.69)	5.85
Secondary outcomes							
Systemic embolism	29/37 208	0.08 (0.05-0.11)	752/2 210 939	0.03 (0.03-0.04)	0.04 (0.02-0.07)	2.29 (1.57-3.34)	4.01
Mesenteric ischemia	<5/37 208^d^	0.00 (0.00-0.02)	26/2 210 894	0	0	3.37 (0.45-25.28)	6.20
Venous thromboembolism	236/36 619	0.64 (0.57-0.73)	8253/2 184 927	0.38 (0.37-0.39)	0.28 (0.20-0.38)	1.75 (1.53-1.99)	2.90
Intracranial extra-axial hemorrhage^e^	196/36 624	0.54 (0.47-0.62)	2233/2 205 200	0.10 (0.10-0.11)	0.40 (0.33-0.48)	4.96 (4.26-5.76)	9.39
Extracranial hemorrhage^f^	584/35 664	1.64 (1.51-1.78)	20 626/2 140 904	0.96 (0.95-0.98)	0.58 (0.45-0.71)	1.60 (1.47-1.74)	2.58
Sudden cardiac death	21/37 208	0.06 (0.04-0.09)	449/2 210 939	0.02 (0.02-0.02)	0.04 (0.02-0.08)	3.08 (1.98-4.81)	5.61
Major ischemic vascular event^g^	1311/35 790	3.66 (3.47-3.87)	45 479/2 129 431	2.14 (2.12-2.16)	1.39 (1.20-1.61)	1.65 (1.56-1.75)	2.69
Major arterial vascular event^h^	1108/36 349	3.05 (2.87-3.23)	38 377/2 153 671	1.78 (1.76-1.80)	1.12 (0.96-1.32)	1.63 (1.54-1.74)	2.64
Major hemorrhagic vascular event^i^	1268/35 123	3.61 (3.42-3.81)	23 854/2 135 639	1.12 (1.10-1.13)	2.30 (2.11-2.50)	3.05 (2.88-3.23)	5.55
Nonvascular death^j^	3322/37 208	8.93 (8.63-9.24)	62 814/2 210 939	2.84 (2.82-2.86)	8.41 (8.01-8.80)	3.96 (3.82-4.10)	7.38
Death from any cause	3521/37 208	9.46 (9.16-9.78)	67 110/2 210 939	3.04 (3.01-3.06)	8.82 (8.42-9.24)	3.90 (3.77-4.04)	7.26

Abbreviations: aHR, adjusted hazard ratio; ICH, intracerebral hemorrhage; MACE, major adverse cardiovascular event.

^a^ Adjusted for sex, age (5-year bands), study period (4- to 5-year bands), baseline comorbidity (separate covariates for each of the following: hypertension, atrial fibrillation, ischemic stroke, myocardial infarction, systemic embolism, peripheral arterial disease, venous thromboembolism, diabetes, chronic renal failure, chronic hepatic diseases, chronic obstructive pulmonary disorder, disorders indicative of high alcohol intake, congestive heart failure, and cancer) and baseline current use of drugs (separate covariates for each of the following drugs and drug classes: low-dose aspirin, clopidogrel, dipyridamole, vitamin K antagonist, direct oral anticoagulant, statins, thiazides and other nonloop diuretics, loop diuretics, β-blockers, calcium channel blockers, angiotensin-converting enzyme inhibitors and angiotensin II receptor blockers, nonsteroidal anti-inflammatory drugs, selective serotonin reuptake inhibitors, and proton pump inhibitors).

^b^ Defined as stroke (ischemic stroke, ICH, or unspecified stroke), myocardial infarction, systemic embolism, or vascular death.

^c^ Death recorded as sudden cardiac death or death within 30 days of any of the following events: stroke (ischemic stroke, ICH, or unspecified stroke), myocardial infarction, systemic embolism, intracranial extra-axial hemorrhage, revascularization procedure, mesenteric ischemia, venous thromboembolism, or extracranial hemorrhage.

^d^ Not reported to preserve anonymity in view of small cell count.

^e^ Subdural hematoma, subarachnoid hemorrhage, or epidural hematoma.

^f^ Gastrointestinal hemorrhage or other major extracranial hemorrhage (for full definition, see Methods).

^g^ Defined as ischemic stroke, unspecified stroke, myocardial infarction, systemic embolism, revascularization procedures, mesenteric ischemia, venous thromboembolism, or sudden cardiac death.

^h^ Defined as ischemic stroke, unspecified stroke, myocardial infarction, systemic embolism, revascularization procedures, mesenteric ischemia, or sudden cardiac death.

^i^ Defined as ICH, intracranial extra-axial hemorrhage, or extracranial hemorrhage.

^j^ Deaths that do not fulfill criteria for vascular death.

Intracerebral hemorrhage was associated with subsequent ischemic (except MI) and hemorrhagic events across strata defined by sex, age, and subgroups (eTables 2 and 3 in the Supplement), with no consistent higher risk in any subgroup defined by comorbidity. Subdistribution HRs of primary outcomes with death as a competing event returned attenuated associations but in the same direction and of roughly similar magnitude as the aHRs (eTable 4 in the Supplement). For primary outcomes we also calculated annual IRs, which, in the ICH cohort, were markedly higher in the first year of follow-up for IS (ICH cohort: IR, 2.16 [95% CI, 1.87-2.51] per 100 person-years; comparison cohort: IR, 0.46 [95% CI, 0.44-0.48] per 100 person-years), ICH (ICH cohort: IR, 2.58 [95% CI, 2.26-2.95] per 100 person-years; comparison cohort: IR, 0.04 [95% CI, 0.04-0.05] per 100 person-years), and major vascular events (ICH cohort: IR, 6.52 [95% CI, 6.01-7.08] per 100 person-years; comparison cohort: IR, 1.16 [95% CI, 1.13-1.20] per 100 person-years), and slightly higher for MI (ICH cohort: IR, 0.81 [95% CI, 0.63-1.03] per 100 person-years; comparison cohort: IR, 0.48 [95% CI, 0.46-0.51] per 100 person-years) (Figure).

**Figure.**
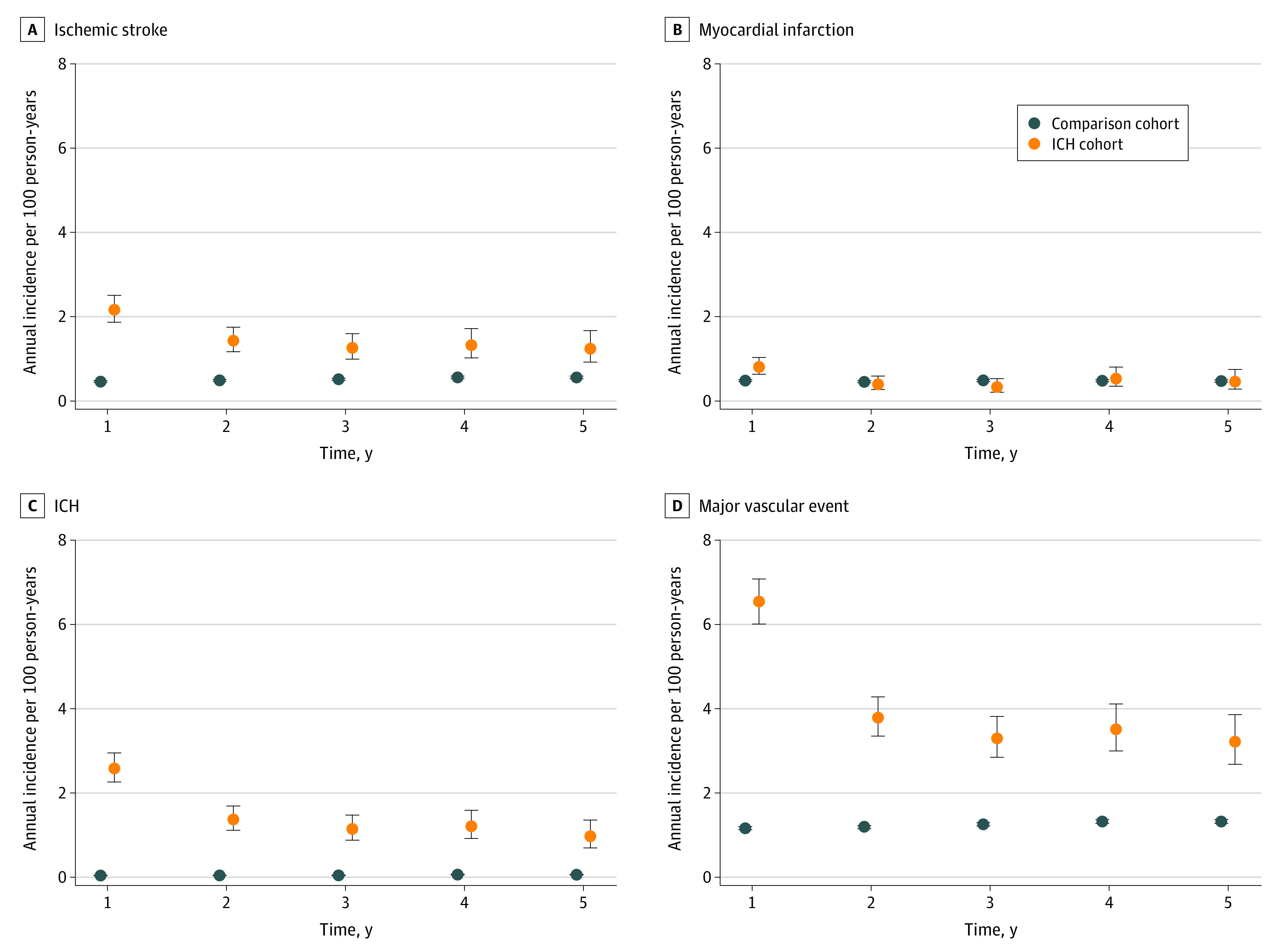
Annual Incidence Rate of Main Outcomes in the First 5 Years of Follow-up of the Intracerebral Hemorrhage (ICH) Cohort and the Comparison Cohort Error bars indicate 95% CIs.

For all secondary outcomes, IRs were higher in the ICH cohort than in the comparison cohort, both for ischemic (eg, major ischemic vascular events: IR, 3.66 [95% CI, 3.47-3.87] per 100 person-years vs 2.14 [95% CI, 2.12-2.16] per 100 person-years; aHR, 1.65 [95% CI, 1.56-1.75]) and hemorrhagic events (eg, major hemorrhagic events: IR, 3.61 [95% CI, 3.42-3.81] per 100 person-years vs 1.12 [95% CI, 1.10-1.13] per 100 person-years; aHR, 3.05 [95% CI, 2.88-3.23]) (Table 2). Incidence rates of vascular death (0.56 [95% CI, 0.48-0.64]) and nonvascular death (8.93 [95% CI, 8.63-9.24]) per 100 person-years were higher in the ICH cohort than in the comparison cohort (0.20 [95% CI, 0.19-0.21] and 2.84 [95% CI, 2.82-2.86], respectively).

The sensitivity analysis (eTable 5 in the Supplement) returned results very similar to the main analysis, except follow-up for death (regardless of other outcomes), which resulted in higher rates than in the main analysis when another outcome preceded death (eg, sensitivity analysis: IR for vascular death in the ICH cohort, 1.27 [95% CI, 1.16-1.38] per 100 person-years; main analysis: IR for vascular death in the ICH cohort, 0.56 [95% CI, 0.48-0.64] per 100 person-years).

### Nested Case-Control Analyses

All individuals in either cohort who experienced a primary outcome were included as cases in the nested case-control analyses for IS (12 987 cases), ICH (1915 cases), MI (10 863 cases), and MACE (30 630 cases). After adjusting for time-varying exposures, the nested case-control analyses showed similar strengths of associations with prior ICH as the cohort analyses (eg, odds ratio, 2.00 [95% CI, 1.82-2.20] for IS and 25.59 [95% CI, 22.30-29.36] for ICH) (Table 3). Subgroup analyses produced findings similar to those in the cohort analyses (eTables 6-9 in the Supplement).

**Table 3.** Nested Case-Control Analysis of the Risk of Primary Outcomes

Outcome during follow-up	No. (%)		OR (95% CI)^a^	Adjusted OR (95% CI)^b^
	Cases	Controls		
Ischemic stroke				
No previous ICH^c^	12 416 (95.6)	213 060 (98.2)	1 [Reference]	1 [Reference]
Previous ICH^d^	571 (4.4)	3825 (1.8)	2.63 (2.40-2.87)	2.00 (1.82-2.20)
ICH				
No previous ICH^c^	1377 (71.9)	63 958 (98.5)	1 [Reference]	1 [Reference]
Previous ICH^d^	538 (28.1)	997 (1.5)	26.65 (23.56-30.16)	25.59 (22.30-29.36)
Myocardial infarction				
No previous ICH^c^	10 669 (98.2)	200 237 (98.2)	1 [Reference]	1 [Reference]
Previous ICH^d^	194 (1.8)	3691 (1.8)	0.98 (0.85-1.14)	0.89 (0.76-1.03)
MACE^e^				
No previous ICH^c^	29 108 (95.0)	264 125 (98.2)	1 [Reference]	1 [Reference]
Previous ICH^d^	1547 (4.9)	4946 (1.8)	2.89 (2.72-3.07)	2.66 (2.49-2.84)

Abbreviations: ICH, intracerebral hemorrhage; MACE, major adverse cardiovascular event; OR, odds ratio.

^a^ Adjusted for age, sex, and index date.

^b^ Adjusted for sex, age, index date, comorbidity (separate covariates for each of the following: hypertension, atrial fibrillation, ischemic stroke, myocardial infarction, systemic embolism, peripheral arterial disease, venous thromboembolism, diabetes, chronic kidney failure, chronic hepatic diseases, chronic obstructive pulmonary disorder, disorders indicative of high alcohol intake, congestive heart failure, and cancer) and current use of drugs (separate covariates for each of the following drugs and drug classes: low-dose aspirin, clopidogrel, dipyridamole, vitamin K antagonist, direct oral anticoagulant, statins, thiazides and other nonloop diuretics, loop diuretics, β-blockers, calcium channel blockers, angiotensin-converting enzyme inhibitors and angiotensin II receptor blockers, nonsteroidal anti-inflammatory drugs, selective serotonin reuptake inhibitors, and proton pump inhibitors). All covariates were determined on the index date and based on all available data (ie, from 10 years before baseline and up to index date).

^c^ Stems from the comparison cohort.

^d^ Stems from the ICH cohort.

^e^ Defined as stroke (ischemic stroke, ICH, or unspecified stroke), myocardial infarction, systemic embolism, or vascular death.

### Severity of Strokes Occurring During Follow-up

In both the ICH cohort and the comparison cohort, ICH during follow-up was associated with more severe strokes and higher case fatality rates than IS (Table 4). For instance, the 30-day case fatality rate in the ICH cohort was 7.2% (95% CI, 5.2%-9.6%) for IS and 28.6% (95% CI, 24.8%-32.6%) for ICH, whereas the 30-day case fatality rate in the comparison cohort was 8.0% (95% CI, 7.5%-8.5%) for IS and 32.0% (95% CI, 29.6%-34.6%) for ICH. The case fatality rate for recurrent ICH was lower in the ICH cohort than for first-ever ICH in the comparison cohort (eg, day 1: 6.7% [95% CI, 4.7%-9.1%] vs 12.0% [95% CI, 10.3%-13.8%]; *P* < .001). Crude SSS scores indicated lower severity of recurrent ICH in the ICH cohort than of ICH in the comparison cohort, albeit overall crude SSS scores did not differ between the groups (median score, 32 [IQR, 12-49] vs 32 [IQR, 12-47]).

**Table 4.** Severity and Outcome of Strokes Occurring During Follow-up

Cohort	Type of stroke during follow-up						*P* value^a^		
	IS		ICH		Unspecified stroke		ICH vs IS in ICH cohort	Recurrent ICH in ICH cohort vs first-ever ICH in comparison cohort	IS in ICH cohort vs IS in comparison cohort
	No.	% (95% CI)	No.	% (95% CI)	No.	% (95% CI)			
**ICH cohort**									
Case fatality rate^b^									
Day 1	NR^c^	NR^c^	36	6.7 (4.7-9.1)	0	0.0 (0.0-7.0)	<.001	.001	.99
Day 7	16	2.8 (1.6-4.5)	96	17.8 (14.7-21.3)	NR^c^	NR^c^	<.001	.01	.60
Day 30	41	7.2 (5.2-9.6)	154	28.6 (24.8-32.6)	5	9.8 (3.3-21.4)	<.001	.14	.48
SSS score, grouped^d^									
Mild (score, 45-58)	272	47.6 (43.5-51.8)	156	29.0 (25.2-33.0)	21	41.2 (27.6-55.8)	<.001	.002	<.001
Moderate (score, 30-44)	147	25.7 (22.2-29.5)	101	18.8 (15.6-22.3)	10	19.6 (9.8-33.1)			
Severe (score, 15-29)	65	11.4 (8.9-14.3)	91	16.9 (13.8-20.4)	NR^c^	NR^c^			
Very severe (score, ≤14)	47	8.2 (6.1-10.8)	136	25.3 (21.7-29.2)	NR^c^	NR^c^			
Missing	40	7.0 (5.1-9.4)	54	10.0 (7.6-12.9)	13	25.5 (14.3-39.6)			
**Comparison cohort**									
Case fatality rate^b^									
Day 1	65	0.5 (0.4-0.7)	165	12.0 (10.3-13.8)	8	1.1 (0.5-2.1)	<.001	NA	NA
Day 7	396	3.2 (2.9-3.5)	320	23.2 (21.0-25.6)	33	4.4 (3.0-6.1)	<.001	NA	NA
Day 30	993	8.0 (7.5-8.5)	441	32.0 (29.6-34.6)	61	8.1 (6.2-10.3)	<.001	NA	NA
SSS score, grouped^d^									
Mild (score, 45-58)	7370	59.4 (58.5-60.2)	416	30.2 (27.8-32.7)	454	60.1 (56.5-63.6)	<.001	NA	NA
Moderate (score, 30-44)	2487	20.0 (19.3-20.7)	283	20.6 (18.4-22.8)	116	15.4 (12.9-18.1)		NA	NA
Severe (score, 15-29)	1090	8.8 (8.3-9.3)	237	17.2 (15.3-19.3)	56	7.4 (5.7-9.5)		NA	NA
Very severe (score, ≤14)	896	7.2 (6.8-7.7)	373	27.1 (24.8-29.5)	42	5.6 (4.0-7.4)		NA	NA
Missing	573	4.6 (4.3-5.0)	68	4.9 (3.9-6.2)	87	11.5 (9.3-14.0)		NA	NA

Abbreviations: ICH, intracerebral hemorrhage; IS, ischemic stroke; NA, not applicable; NR, not reported; SSS, Scandinavian Stroke Scale.

^a^ χ^2^ test.

^b^ Percentage of patients who died within 1, 7, and 30 days of admission for stroke. Includes all deaths (ie, in-hospital and out of hospital). Vital status retrieved from Danish Civil Registration System.

^c^ Not reported to preserve anonymity in view of small cell count.

^d^ Scandinavian Stroke Scale score at time of hospital admission.

## Discussion

In this nationwide study, we found that, compared with controls from the general population, patients with a prior ICH were at increased risk of both hemorrhagic and ischemic vascular events (except MI), associations that were present for both sexes and all age groups and regardless of prior comorbidity. We discovered that the risks of IS, ICH, and MACE were statistically significantly higher in the first year after ICH compared with later years, that ICH occurring during follow-up was more severe and had a higher case fatality rate than IS, and that recurrent ICH had a lower case fatality rate than first-ever ICH.

The higher risk of IS after a prior ICH in the ICH cohort compared with the comparison cohort is in line with previous studies from the US and UK that also used a control group.^5,6,7^ A previous US study based on Medicare data also found an elevated early risk of IS after an ICH.^5^ Although the absolute event rates after an ICH will vary between populations (eg, the event rates after an ICH in Denmark are similar to rates in Oxford, England, but lower than in Lothian, Scotland),^22^ the relative rates between patients with a prior ICH and population controls are similar in Denmark and other countries.^5,6,7^

Taken together, these data identify the first year after an ICH as a particularly high-risk period for both ischemic and hemorrhagic events. This high risk could be due to disability after an ICH, complications of the ICH, withdrawal of secondary prevention agents (such as antithrombotic and cholesterol-lowering agents after an ICH), or low uptake of secondary prevention agents and blood pressure–lowering agents. The first year after an ICH constitutes a window of opportunity for early interventions to reduce the risk of all MACEs with standard and novel approaches to secondary prevention in the short and long term after an ICH.

A recent study from the US^6^ reported a higher risk of MI after an ICH, whereas in our study and a previous large study from the US,^5^ the risk of MI after an ICH did not increase. In a smaller study based on data derived from general practices in the United Kingdom,^7^ the risk of MI after an ICH was also similar to that observed in the population (adjusted subdistribution HR, 1.12 [95% CI, 0.77-1.62]; L. A. García Rodríguez, MD, personal communication document, September 26, 2021). We speculate whether our findings indicate that patients with an ICH have a higher burden of cerebral small vessel disease but not of arteriosclerosis in other vascular beds, a hypothesis that needs to be explored in future studies.

### Strengths and Limitations

Our study has several strengths. We used data from population-based registries with complete coverage and continuously collected data on all Danish residents, thereby eliminating recall bias and minimizing selection bias. Also, loss to follow-up because of emigration was minimal in both cohorts (ie, <0.4%). The codes we used to identify IS,^23^ ICH,^20^ and MI^13^ are reported to be highly accurate (PPVs >80%), and we used a validated algorithm to ascertain recurrent ICH.^20^ We classified stroke severity based on prospectively collected information from the Stroke Registry. The ICH and comparison cohorts were well matched at baseline, and there were few missing data.

Our study also has some limitations. The PPV of stroke diagnosis codes in the Stroke Registry is high.^23^ The registry lacks information on the underlying cause of ICH. However, approximately 80% of ICH cases recorded in the Stroke Registry were spontaneous.^16,24^ The completeness of stroke diagnosis codes in the Stroke Registry is reported to be high (approximately 90% for IS^23^ and approximately 80% for spontaneous ICH^16^), but we cannot exclude some variation in the sensitivity of stroke diagnosis codes over time and across age groups.^16^ To achieve high validity, nonstroke outcomes in our study were identified using primary diagnosis codes for inpatient contacts only, which could underestimate outcomes of mild severity that did not result in hospital admission or outcomes that occurred during a hospital admission for another condition. Furthermore, our choice of diagnosis codes to ascertain vascular death, although in alignment with population-based research in the field,^22^ was conservative. Along with the lack of data on causes of out-of-hospital deaths, this likely led to an underestimation of the rate of vascular death in both the comparison and the ICH cohorts. However, others have also reported that patients with an ICH more frequently die of nonvascular rather than vascular causes.^25^

Although we had data on many risk factors for ischemic events, we lacked data on ICH location, which is associated with long-term risk of recurrent ICH,^22,26,27^ and on other brain imaging biomarkers (eg, ICH volume and markers of small vessel disease) of potential prognostic interest. We did not include information on socioeconomic status, but previous analyses found that the addition of income level and educational level to models similar to those used in this study had little association with risk estimates.^24^ We did not have data on smoking and alcohol use, for which we used proxies, and we lacked data on blood pressure measurements. Therefore, residual confounding by the aforementioned factors and by other insufficiently measured or unmeasured factors is possible. Our E-value estimates indicate that unmeasured confounding would have to be fairly strong to explain the observed associations. Additionally, our data were from people of primarily European ancestry, which limits the generalizability of the study.

## Conclusions

This cohort study suggests that, compared with the general population, patients with a prior ICH in Denmark had statistically significantly higher rates of major ischemic (except MI) and hemorrhagic vascular events, especially in the first year after an ICH, and that the severity and case fatality of recurrent ICH may outweigh those of IS. These findings emphasize the need for further research into the reasons for the higher risks of MACEs in the first year after an ICH and for further research into effective secondary preventive measures to reduce these risks for patients with an ICH.

## Notes

Supplement.
eMethods.
**eTable 1.** Diagnosis and Procedure Codes for Study Covariates Identified in Danish National Patient Registry or Danish National Prescription Registry as Specified **eTable 2.** Crude Incidence Rates of Primary Outcomes During Follow-up, Overall and Stratified by Baseline Comorbidities **eTable 3.** Hazard Ratio of Primary Outcomes and Select Secondary Outcomes in ICH Cohort, Overall and Stratified by Age, Sex, and Baseline Comorbidities (Comparison Cohort Used as Reference) **eTable 4.** Hazard Ratios for Primary Outcomes and Select Secondary Outcomes in ICH Cohort With Comparison Cohort as Reference and Including Estimates That Adjust for Death as Competing Event **eTable 5.** Incidence Rates of Primary and Secondary Study Outcomes Based on Individual Follow-up for Each Outcome With Censoring Only Contingent on Migration, Death, or End of Study Period (as Opposed to Main Analysis, Where First Occurrence of a Primary Outcome Was Also a Censoring Criterion) **eTable 6.** Nested Case-Control Analysis of the Risk of Ischemic Stroke During Follow-up Associated With Having Had an Intracerebral Hemorrhage at Baseline **eTable 7.** Nested Case-Control Analysis of the Risk of Intracerebral Hemorrhage During Follow-up Associated With Having Had an Intracerebral Hemorrhage at Baseline **eTable 8.** Nested Case-Control Analysis of the Risk of Myocardial Infarction During Follow-up Associated With Having Had an Intracerebral Hemorrhage at Baseline **eTable 9.** Nested Case-Control Analysis of the Risk of Major Adverse Cardiovascular Event (Stroke, Myocardial Infarction, Systemic Embolism, or Vascular Death) During Follow-up Associated With Having Had an Intracerebral Hemorrhage at Baseline **eFigure 1.** Flow Chart of Creation of Intracerebral Hemorrhage (ICH) Cohort and Comparison Cohort Based on Data From a Previous Study **eFigure 2.** Cohort Design **eFigure 3.** Nested Case-Control Design **eFigure 4.** Cumulative Incidence of Ischemic Stroke, Intracerebral Hemorrhage, and Myocardial Infarction **eFigure 5.** Cumulative Incidence of Major Adverse Cardiovascular Event (Stroke, Myocardial Infarction, Systemic Embolism, or Vascular Death) and All-Cause Death **eFigure 6.** Cumulative Incidence of Ischemic Stroke, Intracerebral Hemorrhage, and Major Adverse Cardiovascular Event Stratified by Comorbid Atrial Fibrillation
eReferences.
