# Supplementary material for: Association of Prior Intracerebral Hemorrhage With Major Adverse Cardiovascular Events
Source: JAMA Netw Open. 2022 Oct 3;5(10):e2234215. doi: 10.1001/jamanetworkopen.2022.34215 (PMC9530971; doi:10.1001/jamanetworkopen.2022.34215)
Supplement: Supplement. — eMethods. eTable 1. Diagnosis and Procedure Codes for Study Covariates Identified in Danish National Patient Registry or Danish National Prescription Registry as Specified eTable 2. Crude Incidence Rates of Primary Outcomes During Follow-up, Overall and Stratified by Baseline Comorbidities eTable 3. Hazard Ratio of Primary Outcomes and Select Secondary Outcomes in ICH Cohort, Overall and Stratified by Age, Sex, and Baseline Comorbidities (Comparison Cohort Used as Reference) eTable 4. Hazard Ratios for Primary Outcomes and Select Secondary Outcomes in ICH Cohort With Comparison Cohort as Reference and Including Estimates That Adjust for Death as Competing Event eTable 5. Incidence Rates of Primary and Secondary Study Outcomes Based on Individual Follow-up for Each Outcome With Censoring Only Contingent on Migration, Death, or End of Study Period (as Opposed to Main Analysis, Where First Occurrence of a Primary Outcome Was Also a Censoring Criterion) eTable 6. Nested Case-Control Analysis of the Risk of Ischemic Stroke During Follow-up Associated With Having Had an Intracerebral Hemorrhage at Baseline eTable 7. Nested Case-Control Analysis of the Risk of Intracerebral Hemorrhage During Follow-up Associated With Having Had an Intracerebral Hemorrhage at Baseline eTable 8. Nested Case-Control Analysis of the Risk of Myocardial Infarction During Follow-up Associated With Having Had an Intracerebral Hemorrhage at Baseline eTable 9. Nested Case-Control Analysis of the Risk of Major Adverse Cardiovascular Event (Stroke, Myocardial Infarction, Systemic Embolism, or Vascular Death) During Follow-up Associated With Having Had an Intracerebral Hemorrhage at Baseline eFigure 1. Flow Chart of Creation of Intracerebral Hemorrhage (ICH) Cohort and Comparison Cohort Based on Data From a Previous Study eFigure 2. Cohort Design eFigure 3. Nested Case-Control Design eFigure 4. Cumulative Incidence of Ischemic Stroke, Intracerebral Hemorrhage, and Myocardial Infarction eFigure 5. Cumul [file jamanetwopen-e2234215-s001.pdf]

# Supplementary Online Content

Gaist D, Hald SM, García Rodríguez LA, et al. Association of prior intracerebral hemorrhage with major adverse cardiovascular events. *JAMA Netw Open.* 2022;5(10):e2234215. doi:10.1001/jamanetworkopen.2022.34215

**eMethods.**
**eTable 1.** Diagnosis and Procedure Codes for Study Covariates Identified in Danish National Patient Registry or Danish National Prescription Registry as Specified
**eTable 2.** Crude Incidence Rates of Primary Outcomes During Follow-up, Overall and Stratified by Baseline Comorbidities
**eTable 3.** Hazard Ratio of Primary Outcomes and Select Secondary Outcomes in ICH Cohort, Overall and Stratified by Age, Sex, and Baseline Comorbidities (Comparison Cohort Used as Reference)
**eTable 4.** Hazard Ratios for Primary Outcomes and Select Secondary Outcomes in ICH Cohort With Comparison Cohort as Reference and Including Estimates That Adjust for Death as Competing Event
**eTable 5.** Incidence Rates of Primary and Secondary Study Outcomes Based on Individual Follow-up for Each Outcome With Censoring Only Contingent on Migration, Death, or End of Study Period (as Opposed to Main Analysis, Where First Occurrence of a Primary Outcome Was Also a Censoring Criterion)
**eTable 6.** Nested Case-Control Analysis of the Risk of Ischemic Stroke During Follow-up Associated With Having Had an Intracerebral Hemorrhage at Baseline
**eTable 7.** Nested Case-Control Analysis of the Risk of Intracerebral Hemorrhage During Follow-up Associated With Having Had an Intracerebral Hemorrhage at Baseline
**eTable 8.** Nested Case-Control Analysis of the Risk of Myocardial Infarction During Follow-up Associated With Having Had an Intracerebral Hemorrhage at Baseline
**eTable 9.** Nested Case-Control Analysis of the Risk of Major Adverse Cardiovascular Event (Stroke, Myocardial Infarction, Systemic Embolism, or Vascular Death) During Follow-up Associated With Having Had an Intracerebral Hemorrhage at Baseline
**eFigure 1.** Flow Chart of Creation of Intracerebral Hemorrhage (ICH) Cohort and Comparison Cohort Based on Data From a Previous Study
**eFigure 2.** Cohort Design
**eFigure 3.** Nested Case-Control Design
**eFigure 4.** Cumulative Incidence of Ischemic Stroke, Intracerebral Hemorrhage, and Myocardial Infarction
**eFigure 5.** Cumulative Incidence of Major Adverse Cardiovascular Event (Stroke, Myocardial Infarction, Systemic Embolism, or Vascular Death) and All-Cause Death
**eFigure 6.** Cumulative Incidence of Ischemic Stroke, Intracerebral Hemorrhage, and Major Adverse Cardiovascular Event Stratified by Comorbid Atrial Fibrillation
**eReferences.**

This supplementary material has been provided by the authors to give readers additional information about their work.

## eMethods.

### Creation of cohorts for present study based on data from previous study

In a previous study[1] we used the Danish Stroke Registry[2] (Stroke Registry) to identify 16,765 patients with first-ever intracerebral hemorrhage (ICH) admitted in 2005-2018 in Denmark. We matched each of these cases on age, sex, and index date (date of admission of case) to 660,477 general population controls.[1] The index date from the previous study is referred to here as inclusion date to avoid confusion with the nomenclature used in the nested case-control analysis planned as part of the present study (see main text). In the previous case-control study, we used risk-set sampling and controls were replaced after selection. This meant that individuals could be included as controls more than once and also that individuals sampled as controls could later become cases. In this study we defined the ICH-cohort and the comparison cohort to be mutually exclusive (i.e., individuals contributed only to one cohort). Therefore, we identified the chronologically first status of each individual and classified individuals accordingly as belonging to the ICH cohort or the comparison cohort (eFigure 1). In accordance with how we defined the source population (see main text), we excluded individuals aged <45 years on inclusion date (as ICH in this age-group is more likely to be non-spontaneous and ischemic events are less likely to occur in ICH survivors); and patients from the ICH-cohort who died within 30 days of their inclusion date (~30%) as well as patients from the comparison cohort who died within 30 days of their respective inclusion date (eFigure 1). For cohort members who survived the first 30 days, we ignored admissions with diagnosis codes corresponding to major vascular events recorded on day 0 to 30 post-inclusion date (i.e., we applied a 30-day blanking period). This was based on results of a previous validation study, where we found the positive predictive value (PPV) of a diagnosis of recurrent spontaneous symptomatic ICH (re-ICH) to be very low if the first 30-day period after an ICH is included. Conversely, if a 30-day blanking period is used, the PPV for re-ICH in DSR is high (90%) with an acceptable sensitivity (76%).[3] Consequently, the start date for the present study was 31 days after the inclusion date.

### Assessment of exposure to medications

Information on prescriptions dispensed at community pharmacies in Denmark has been prospectively recorded in the Danish National Prescription Registry[4] (Prescription Registry) since 1995. For each prescription, the date of dispensing and a full account of the dispensed product, including the anatomical therapeutic chemical[5] (ATC) code, are recorded. The indication and prescribed dose are not available in the Prescription Registry.

To calculate date of end of supply of each prescription, we set each prescription to last the number of days that corresponded to the number of pills dispensed (for dipyridamole, ticagrelor, dabigatran, apixaban, and all nonsteroidal anti-inflammatory drugs divided by a factor of 2; and for warfarin divided by a factor of 1.5[6]); however, for proton pump inhibitors (PPI) we instead used the defined daily dose[5] (DDD; 1 DDD corresponds to 20 mg omeprazole, 40mg pantoprazole, 30mg lansoprazole, 20mg rabeprazole, 30mg esomeprazole).

After calculating the length of each supply as described above, we calculated episodes of treatment.

We did this based on prescriptions for drugs in the 10-year period up to inclusion date for the cohort analysis (eFigure 2). Between pairs of consecutive prescriptions, we allowed a grace period of 60 days between the end of supply of the first prescription of that pair and the date of the next prescription. Based on the most recent treatment episode before the inclusion date, we divided exposure by recency into current use (prescription supply covered the inclusion date or ended less than 30 days before the inclusion date), past use (prescription supply ended 31-365 days before inclusion date), and non-use (no recorded prescription, or prescription supply ended more than 365 days before inclusion date). A treatment episode for a drug or drug class comprised the length of consecutive prescriptions allowing for gaps between prescriptions of 60 days or less (grace period).

For the nested case-control analysis we used the same approach but with the index date as the reference point (as opposed to inclusion date in the cohort analysis) (eFigure 3). Using both the exposure history in the 10-year period before inclusion date and exposures to drugs during follow-up and based on the most recent treatment episode before the index date, we divided exposure by recency into current use (prescription supply covered the index date or ended less than 30 days before the index date), past use (prescription supply ended 31-365 days before index date), and non-use (no recorded prescription, or prescription supply ended more than 365 days before index date).

### Statistical analysis

*Nested case-control analysis*

We performed subgroup analyses for sex, age (<75 years; ≥75 years), atrial fibrillation (AF), diabetes (DM), history of ischemic stroke (IS), history of major ischemic vascular event (including IS), and history of major ischemic vascular event other than IS. In these analyses, AF and DM were classified based on all available prior information, while history of major ischemic vascular events, IS, and ICH were classified based on information at baseline (cohort entry). For the subgroup analyses, we re-sampled cases and controls to achieve comparisons of e.g., cases with AF and controls with AF on index date. For the subgroup analyses, we used unconditional logistic regression and included the same covariates as in the main case-control analyses, in addition to covariates for sex, age (5-year bands), index date year (3-year bands), and duration of follow-up (4-year bands).

© 2022 Gaist D et al. *JAMA Network Open.*

**eTable 1.** Diagnosis and Procedure Codes for Study Covariates Identified in Danish National Patient Registry or Danish National Prescription Registry as Specified

| | **Diagnosis code (ICD-10) or procedure code (sksube) from Danish National Patient Registry (unless otherwise specified)** | **Drug (ATC) code** |
|---|---|---|
| **Primary outcomes**<br>*For diagnosis codes inpatient hospital contacts with primary position codes only (unless otherwise specified)* | | |
| 1. Ischemic stroke | I63 [in Danish Stroke Registry] | |
| 2. Intracerebral hemorrhage | I61 [in Danish Stroke Registry] | |
| 3. Myocardial infarction | I21, I22, I23 | |
| 4. Major vascular event | | |
| 4a. Stroke | | |
| 4a1. Ischemic stroke | I63 [in Danish Stroke Registry] | |
| 4a2. Intracerebral hemorrhage | I61 [in Danish Stroke Registry] | |
| 4a3. Stroke of unspecified type | I64 [in Danish Stroke Registry] | |
| 4b. Myocardial infarction | I21, I22, I23 | |
| 4c. Systemic embolism | I74 | |
| 4d. Vascular death – Death within 30 days if a hospital admission for one or more of the following events: | | |
| 4d1. Stroke | | |
| 4d1a. Intracerebral hemorrhage | I61 [in Danish Stroke Registry] | |
| 4d1b. Ischemic stroke | I63 [in Danish Stroke Registry] | |
| 4d1c. Stroke of unspecified type | I64 [in Danish Stroke Registry] | |
| Any of the following non-stroke events: | | |
| 4d2. Intracranial extraaxial hemorrhage | | |
| 4d2a. Subdural hematoma | S065, I620 | |
| 4d2b. Subarachnoid hemorrhage | I60 | |
| 4d2c. Epidural hematoma | I621 | |
| 4d2d. Intracranial unspecified | I629 | |
| 4d3. Myocardial infarction | I21, I22, I23 | |
| 4d4. Systemic embolism | I74 | |
| 4d5. Revascularization procedures | | |

© 2022 Gaist D et al. *JAMA Network Open.*

| | | |
|---|---|---|
| 4d5a. Thrombolysis (any organ)[1] | BOHA1 (sksube) | |
| 4d5b. Percutaneous coronary intervention[1] [includes stenting] | KFNG, KFNF (sksube) | |
| 4d5c. Coronary artery bypass grafting | KFNA, KFNB, KFNC, KFND, KFNE, KFNH20 (sksube) | |
| 4d5d. Carotid artery procedures (includes all arteries derived from aortic arch and its branches)[2] | KPAF (endarterectomy), KPAE (thrombectomy) (sksube) | |
| 4d4e. Visceral artery, thrombectomy/embolectomy (mesenteric, renal, visceral)[2] | KPCE, KPCF (sksube) | |
| 4d4f. Lower limb revascularization[3] (thrombectomy/embolectomy, stents, bypass surgery etc) | KPDE, KPDF, KPDH, KPDN, KPDP, KPDQ, KPEE, KPEF, KPEH, KPEN, KPEP, KPEQ, KPFE, KPFH, KPFN, KPFP, KPFQ, KPDU74, KPDU84 (sksube) | |
| 4d6. Mesenteric ischemia | K550E K550H | |
| 4d7. Sudden cardiac death | I461 | |
| 4d8. Venous thromboembolism (DVT/PE) | I26, I801, I802, I803, I808, I809 | |
| 4d9. Extracranial hemorrhage | | |
| 4d9a. Gastrointestinal bleed (upper, lower, or unspecified) | K228F, K250, K252, K254, K256, K260, K262, K638B, K264, K266, K270, K272, K274, K276, K280, K282, K284, K286, K290, K298A, K625, K638B, K638C, K290-K292, K920, K921, K922 | |
| 4d9b. Esophageal varicose vein hemorrhage or esophageal hemorrhage | I850, I864A, K228F | |
| 4d9c. Hematuria | N02, R31 | |
| 4d9d. Hemopericardium | I312 | |
| 4d9e. Peritoneal hemorrhage | K661 | |
| 4d9f. Hemothorax | J942 | |
| 4d9g. Hemorrhage in bile duct or pancreas | K838F, K868G | |
| 4d9h. Hemorrhage in spinal cord | G951A | |
| 4d9i. Acute bleeding anemia | D62 | |
| **Secondary outcomes** | | |

© 2022 Gaist D et al. *JAMA Network Open.*

| *For diagnosis codes inpatient hospital contacts with primary position codes only (unless otherwise specified)* | | |
|---|---|---|
| 1. Stroke | | |
| 1a. Ischemic stroke | I63 [in Danish Stroke Registry] | |
| 1b. Intracerebral hemorrhage | I61 [in Danish Stroke Registry] | |
| 1c. Stroke of uncertain type | I64 [in Danish Stroke Registry] | |
| 2. Intracranial extraaxial hemorrhage | | |
| 2a.Subdural | S065, I620 | |
| 2b.Subarachnoid | I60 | |
| 2c. Epidural | I621 | |
| 2d. Intracranial unspecified | I629 | |
| 3. Systemic embolism | I74 | |
| 4. Mesenteric ischemia | K550E K550H | |
| 5. Venous thromboembolism (DVT or PE) | I26, I801, I802, I803, I808, I809 | |
| 6. Extracranial hemorrhage | | |
| 6a. Gastrointestinal bleed (upper, lower, or unspecified) | K228F, K250, K252, K254, K256, K260, K262, K638B, K264, K266, K270, K272, K274, K276, K280, K282, K284, K286, K290, K298A, K625, K638B, K638C, K290-K292, K920, K921, K922 | |
| 6b. Esophageal varicose vein hemorrhage or esophageal hemorrhage | I850, I864A, K228F | |
| 6c. Hematuria | N02, R31 | |
| 6d. Hemopericardium | I312 | |
| 6e. Peritoneal hemorrhage | K661 | |
| 6f. Hemothorax | J942 | |
| 6g. Hemorrhage in bile duct or pancreas | K838F, K868G | |
| 6h. Hemorrhage in spinal cord | G951A | |
| 6i. Acute bleeding anemia | D62 | |
| 7. Sudden cardiac death | I461 | |
| 8. Major adverse cardiovascular event | | |
| 8a. Ischemic stroke | I63 [in Danish Stroke Registry] | |
| 8b. Stroke of uncertain type | I64 [in Danish Stroke Registry] | |

© 2022 Gaist D et al. *JAMA Network Open.*

| | | |
|---|---|---|
| 8c. Myocardial infarction | I21, I22, I23 | |
| 8d. Systemic embolism | I74 | |
| 8e. Revascularization procedures | | |
| 8e1. Thrombolysis (any organ)[1] | BOHA1 (sksube) | |
| 8e2. Percutaneous coronary intervention[1] [includes stenting] | KFNG, KFNF (sksube) | |
| 8e3. Coronary artery bypass grafting | KFNA, KFNB, KFNC, KFND, KFNE, KFNH20 (DNPR, sksube) | |
| 8e4. Carotid artery procedures (includes all arteries derived from aortic arch and its branches)[2] | KPAF (endarterectomy), KPAE (thrombectomy) (sksube) | |
| 8e5. Visceral artery, thrombectomy/embolectomy (mesenteric, renal, visceral)[2] | KPCE, KPCF (sksube) | |
| 8e6. Lower limb revascularization[3] (thrombectomy/embolectomy, stents, bypass surgery etc) | KPDE, KPDF, KPDH, KPDN, KPDP, KPDQ, KPEE, KPEF, KPEH, KPEN, KPEP, KPEQ, KPFE, KPFH, KPFN, KPFP, KPFQ, KPDU74, KPDU84 (sksube) | |
| 8f. Mesenteric ischemia | K550E K550H | |
| 8g. Venous thrombosis (DVT or PE) | I26, I801, I802, I803, I808, I809 ( | |
| 8h. Sudden cardiac death | I461 | |
| 9. Major hemorrhagic vascular event | | |
| 9a. ICH | I61 [in Danish Stroke Registry] | |
| 9b. Extracranial hemorrhage | | |
| 9b1. Gastrointestinal bleed (upper, lower, or unspecified) | K228F, K250, K252, K254, K256, K260, K262, K638B, K264, K266, K270, K272, K274, K276, K280, K282, K284, K286, K290, K298A, K625, K638B, K638C, K290-K292, K920, K921, K922 | |
| 9b2. Esophageal varicose vein hemorrhage or esophageal hemorrhage | I850, I864A, K228F | |
| 9b3. Hematuria | N02, R31 | |
| 9b4. Hemopericardium | I312 | |
| 9b5. Peritoneal hemorrhage | K661 | |
| 9b6. Hemothorax | J942 | |

© 2022 Gaist D et al. *JAMA Network Open.*

| | | |
|---|---|---|
| 9b7. Hemorrhage in bile duct or pancreas | K838F, K868G | |
| 9b8. Hemorrhage in spinal cord | G951A | |
| 9b9. Acute bleeding anemia | D62 | |
| 9c. Intracranial extraaxial hemorrhage | | |
| 9c1. Subdural | S065, I620 | |
| 9c2. Subarachnoid | I60 | |
| 9c3. Epidural | I621 | |
| 9c4. Intracranial unspecified | I629 | |
| 10. Major adverse cardiovascular event | | |
| 10a. Ischemic stroke | I63 [in Danish Stroke Registry] | |
| 10b. Stroke of uncertain type | I64 [in Danish Stroke Registry] | |
| 10c. Myocardial infarction | I21, I22, I23 | |
| 10d. Systemic embolism | I74 | |
| 10e. Revascularization procecdures | | |
| 10e1. Thrombolysis (any organ)[1] | BOHA1 (sksube) | |
| 10e2. Percutaneous coronary intervention[1] [includes stenting] | KFNG, KFNF (sksube) | |
| 10e3. Coronary artery bypass grafting | KFNA, KFNB, KFNC, KFND, KFNE, KFNH20 (sksube) | |
| 10e4. Carotid artery procedures (includes all arteries derived from aortic arch and its branches)[2] | KPAF (endarterectomy), KPAE (thrombectomy) (sksube) | |
| 10e5. Visceral artery, thrombectomy/embolectomy (mesenteric, renal, visceral)[2] | KPCE, KPCF (sksube) | |
| 10e6. Lower limb revascularization[3] (thrombectomy/embolectomy, stents, bypass surgery etc) | KPDE, KPDF, KPDH, KPDN, KPDP, KPDQ, KPEE, KPEF, KPEH, KPEN, KPEP, KPEQ, KPFE, KPFH, KPFN, KPFP, KPFQ, KPDU74, KPDU84 (sksube) | |
| 10f. Mesenteric ischemia | K550E K550H | |
| 10g. Sudden cardiac death | I461 | |
| 11. All-cause death | | |
| 12. Vascular death – Death within 30 days of hospital admission for one or more of the following events: | | |

© 2022 Gaist D et al. *JAMA Network Open.*

| | | |
|---|---|---|
| 12a. Stroke | | |
| 12a1. Intracerebral hemorrhage | I61 [in Danish Stroke Registry] | |
| 12a2. Ischemic stroke | I63 [in Danish Stroke Registry] | |
| 12a3. Stroke of uncertain type | I64 [in Danish Stroke Registry] | |
| Any of the following non-stroke events: | | |
| 12b. Intracranial extraaxial hemorrhage | | |
| 12b1. Subdural | S065, I620 | |
| 12b2. Subarachnoid | I60 | |
| 12b3. Epidural | I621 | |
| 12b4. Intracranial unspecified | I629 | |
| 12c. Myocardial infarction | I21, I22, I23 | |
| 12d. Systemic embolism | I74 | |
| 12e. Revascularization procedures | | |
| 12e1. Thrombolysis (any organ)[1] | BOHA1 (sksube) | |
| 12e2. Percutaneous coronary intervention[1] [includes stenting] | KFNG, KFNF (sksube) | |
| 12e3. Coronary artery bypass grafting | KFNA, KFNB, KFNC, KFND, KFNE, KFNH20 (sksube) | |
| 12e4. Carotid artery procedures (includes all arteries derived from aortic arch and its branches)[2] | KPAF (endarterectomy), KPAE (thrombectomy) (sksube) | |
| 12e5. Visceral artery, thrombectomy/embolectomy (mesenteric, renal, visceral)[2] | KPCE, KPCF (sksube) | |
| 12e6. Lower limb revascularization[3] (thrombectomy/embolectomy, stents, bypass surgery etc) | KPDE, KPDF, KPDH, KPDN, KPDP, KPDQ, KPEE, KPEF, KPEH, KPEN, KPEP, KPEQ, KPFE, KPFH, KPFN, KPFP, KPFQ, KPDU74, KPDU84 (sksube) | |
| 12f. Mesenteric ischemia | K550E K550H | |
| 12g. Sudden cardiac death | I461 | |
| 12h. Venous thromboembolism (DVT or PE) | I26, I801, I802, I803, I808, I809 | |
| 12i. Extracranial hemorrhage | | |
| 12i1.Gastrointestinal bleed (upper, lower, or unspecified) | K228F, K250, K252, K254, K256, K260, K262, K638B, K264, K266, K270, K272, K274, K276, K280, K282, K284, K286, K290, K298A, | |

© 2022 Gaist D et al. *JAMA Network Open.*

| | | |
|---|---|---|
| | K625, K638B, K638C, K290-K292, K920, K921, K922 | |
| 12i2. Esophageal varicose vein hemorrhage or esophageal hemorrhage | I850, I864A, K228F | |
| 12i3. Hematuria | N02, R31 | |
| 12i4. Hemopericardium | I312 | |
| 12i5. Peritoneal hemorrhage | K661 | |
| 12i6. Hemothorax | J942 | |
| 12i7. Hemorrhage in bile duct or pancreas | K838F, K868G | |
| 12i8. Hemorrhage in spinal cord | G951A | |
| 12i9. Acute bleeding anemia | D62 | |
| 13. Non-vascular death (death not classified as vascular death) | | |
| **Covariates – Disorders**<br>*For diagnosis codes: Inpatient or outpatient, primary or secondary position (unless otherwise specified)* | | |
| Hypertension | I10-I15 | C03A, C08CA, C08DB01, C09A, C09B, C09C, C09D |
| Ischemic stroke | I63 [in Danish Stroke Registry] | |
| Myocardial infarction | I21, I22, I23 | |
| Systemic embolism | I74 | |
| Peripheral arterial disease | I702, I739 | |
| Venous thromboembolism (DVT/PE) | I26, I801, I802, I803, I808, I809 | |
| Diabetes | E10-E14 | A10 |
| Chronic kidney failure | N18 (excluding N181), N19, Z992, Z940 | |
| Chronic hepatic diseases | K71-K77 | |
| Atrial fibrillation | I48 | |
| Congestive heart failure | I110, I130, I132, I420, I426, I427, I428, I429, I50 | |
| Cancer | C00-C97 (C44 not included) | |
| Disorders/events or drug use indicative of alcohol misuse | E244, F10, G312, G621, G721, I426, K292, K70, K860, T510, T519, Z502, Z714, Z721, | N07BB |

© 2022 Gaist D et al. *JAMA Network Open.*

| | | |
|---|---|---|
| Chronic obstructive pulmonary disorder (COPD) | J42, J42, J44 | R03 [in subjects aged 45+ years] |
| **Covariates – use of medications** | | |
| Antiplatelet drugs | | |
| Aspirin – low dose | | B01AC06 – acetylsalicylic acid (75 mg, 100 mg, or 150 mg per tablet)<br>B01AC30 - acetylsalicylic acid (50 mg per tablet) in combination with dipyridamole |
| Clopidogrel | | B01AC04 (75 mg per tablet) |
| Dipyridamole | | B01AC07 (100 mg per tablet, or 200 mg per tablet) |
| Other ADP-receptor blockers | | B01AC22 – prasugrel (5 mg, or 10 mg per tablet)<br>B01AC24 – ticagrelor (60 mg, or 90 mg per tablet) |
| Anticoagulant drugs | | |
| Vitamin K antagonists | | B01AA |
| Direct oral anticoagulants (DOAC) | | |
| Dabigratran | | B01AE07 |
| Rivaroxaban | | B01AF01 |
| Apixaban | | B01AF02 |
| Edoxaban | | B01AF03 |
| Drugs with antihypertensive effects[a] | | |
| Thiazides and other non-loop diuretics | | C03A, C03D, C03E |
| Loop diuretics | | C03C |
| Beta-blockers | | C07 |
| Calcium channel blockers | | C08 |
| ACE inhibitors and Angiotensin II receptor blockers (plain or in combinations) | | C09A, C09B, C09C, C09D |
| Statins | | C10AA |

© 2022 Gaist D et al. *JAMA Network Open.*

| | | |
|---|---|---|
| Selective serotonin reuptake inhibitors | | N06AB |
| Nonsteroidal anti-inflammatory drugs | | M01A (including Cox2 inhibitors), excluding M01AX |
| Proton pump inhibitors | | A02BC |

[a]Indication for drug use not available; some of the drugs may have been used for indications other than hypertension. Note that ‘ever use’ of some of these drugs was included in the definition of the hypertension covariate (i.e., C03A, C08CA, C08DB01, C09A, C09B, C09C, C09D).

© 2022 Gaist D et al. *JAMA Network Open.*

**eTable 2.** Crude Incidence Rates of Primary Outcomes During Follow-up, Overall and Stratified by Baseline Comorbidities

| | **Ischemic stroke** | | | **Myocardial infarct** | | | **Intracerebral hemorrhage** | | | **Major adverse cardiovascular events**[1] | | |
|---|---|---|---|---|---|---|---|---|---|---|---|---|
| **Baseline characteristic /comorbidity** | Person-years | No. of events | Event rate per 100 pyrs (95% CI) | Person-years | No. of events | Event rate per 100 pyrs (95% CI) | Person-years | No. of events | Event rate per 100 pyrs (95% CI) | Person-years | No. of events | Event rate per 100 pyrs (95% CI) |
| **All** | | | | | | | | | | | | |
| ICH-cohort | 37,482 | 571 | 1.52 (1.40-1.65) | 37,482 | 194 | 0.52 (0.45-0.60) | 37,482 | 538 | 1.44 (1.32-1.56) | 37,208 | 1,547 | 4.16 (3.96-4.37) |
| Comparison cohort | 2,216,351 | 12,416 | 0.56 (0.55-0.57) | 2,216,351 | 10,669 | 0.48 (0.47-0.49) | 2,216,351 | 1,377 | 0.06 (0.06-0.07) | 2,210,939 | 29,780 | 1.35 (1.33-1.36) |
| **Male sex** | | | | | | | | | | | | |
| ICH-cohort | 20,829 | 319 | 1.53 (1.37-1.71) | 20,829 | 119 | 0.57 (0.48-0.68) | 20,829 | 266 | 1.28 (1.13-1.44) | 20,664 | 844 | 4.08 (3.82-4.37) |
| Comparison cohort | 1,193,409 | 6,881 | 0.58 (0.56-0.59) | 1,193,409 | 6,933 | 0.58 (0.57-0.59) | 1,193,409 | 707 | 0.06 (0.06-0.06) | 1,190,362 | 17,505 | 1.47 (1.45-1.49) |
| **Female sex** | | | | | | | | | | | | |
| ICH-cohort | 16,654 | 252 | 1.51 (1.34-1.71) | 16,654 | 75 | 0.45 (0.36-0.56) | 16,654 | 272 | 1.63 (1.45-1.84) | 16,544 | 703 | 4.25 (3.95-4.58) |
| Comparison cohort | 1,022,943 | 5,535 | 0.54 (0.53-0.56) | 1,022,943 | 3,736 | 0.37 (0.35-0.38) | 1,022,943 | 670 | 0.07 (0.06-0.07) | 1,020,577 | 12,275 | 1.20 (1.18-1.22) |
| **Age 45-59 years** | | | | | | | | | | | | |

© 2022 Gaist D et al. *JAMA Network Open.*

| | Ischemic stroke | | | Myocardial infarct | | | Intracerebral hemorrhage | | | Major adverse cardiovascular events[1] | | |
|---|---|---|---|---|---|---|---|---|---|---|---|---|
| ICH-cohort | 10,047 | 100 | 1.00 (0.82-1.21) | 10,047 | 43 | 0.43 (0.32-0.58) | 10,047 | 75 | 0.75 (0.60-0.94) | 9,999 | 247 | 2.47 (2.18-2.80) |
| Comparison cohort | 499,588 | 1,029 | 0.21 (0.19-0.22) | 499,588 | 1,298 | 0.26 (0.25-0.27) | 499,588 | 97 | 0.02 (0.02-0.02) | 499,025 | 2,680 | 0.54 (0.52-0.56) |
| **Age 60-74 years** | | | | | | | | | | | | |
| ICH-cohort | 17,160 | 248 | 1.45 (1.28-1.64) | 17,160 | 77 | 0.45 (0.36-0.56) | 17,160 | 247 | 1.44 (1.27-1.63) | 17,010 | 655 | 3.85 (3.57-4.16) |
| Comparison cohort | 966,747 | 4,791 | 0.50 (0.48-0.51) | 966,747 | 4,317 | 0.45 (0.43-0.46) | 966,747 | 481 | 0.05 (0.05-0.05) | 964,582 | 11,145 | 1.16 (1.13-1.18) |
| **Age 75-84 years** | | | | | | | | | | | | |
| ICH-cohort | 8,118 | 178 | 2.19 (1.89-2.54) | 8,118 | 54 | 0.67 (0.51-0.87) | 8,118 | 176 | 2.17 (1.87-2.51) | 8,048 | 512 | 6.36 (5.83-6.94) |
| Comparison cohort | 564,451 | 4,885 | 0.87 (0.84-0.89) | 564,451 | 3,818 | 0.68 (0.66-0.70) | 564,451 | 625 | 0.11 (0.10-0.12) | 562,369 | 11,720 | 2.08 (2.05-2.12) |
| **Age 85+ years** | | | | | | | | | | | | |
| ICH-cohort | 2,157 | 45 | 2.09 (1.56-2.79) | 2,157 | 20 | 0.93 (0.60-1.44) | 2,157 | 40 | 1.85 (1.36-2.53) | 2,152 | 133 | 6.18 (5.21-7.33) |
| Comparison cohort | 185,566 | 1,711 | 0.92 (0.88-0.97) | 185,566 | 1,236 | 0.67 (0.63-0.70) | 185,566 | 174 | 0.09 (0.08-0.11) | 184,963 | 4,235 | 2.29 (2.22-2.36) |

© 2022 Gaist D et al. *JAMA Network Open.*

| | Ischemic stroke | | | Myocardial infarct | | | Intracerebral hemorrhage | | | Major adverse cardiovascular events[1] | | |
|---|---|---|---|---|---|---|---|---|---|---|---|---|
| **Atrial Fibrillation[2]** | | | | | | | | | | | | |
| **Yes** | | | | | | | | | | | | |
| ICH-cohort | 4,015 | 114 | 2.84 (2.36-3.41) | 4,015 | 30 | 0.75 (0.52-1.07) | 4,015 | 59 | 1.47 (1.14-1.90) | 3,947 | 251 | 6.36 (5.62-7.20) |
| Comparison cohort | 133,293 | 1,254 | 0.94 (0.89-0.99) | 133,293 | 845 | 0.63 (0.59-0.68) | 133,293 | 167 | 0.13 (0.11-0.15) | 132,759 | 3,028 | 2.28 (2.20-2.36) |
| **No** | | | | | | | | | | | | |
| ICH-cohort | 33,467 | 457 | 1.37 (1.25-1.50) | 33,467 | 164 | 0.49 (0.42-0.57) | 33,467 | 479 | 1.43 (1.31-1.57) | 33,261 | 1,296 | 3.90 (3.69-4.11) |
| Comparison cohort | 2,083,059 | 11,162 | 0.54 (0.53-0.55) | 2,083,059 | 9,824 | 0.47 (0.46-0.48) | 2,083,059 | 1,210 | 0.06 (0.05-0.06) | 2,078,180 | 26,752 | 1.29 (1.27-1.30) |
| **Hx of Ischemic stroke[2]** | | | | | | | | | | | | |
| **Yes** | | | | | | | | | | | | |
| ICH-cohort | 4,400 | 118 | 2.68 (2.24-3.21) | 4,400 | 19 | 0.43 (0.28-0.68) | 4,400 | 61 | 1.39 (1.08-1.78) | 4,351 | 234 | 5.38 (4.73-6.11) |
| Comparison cohort | 54,227 | 782 | 1.44 (1.34-1.55) | 54,227 | 329 | 0.61 (0.54-0.68) | 54,227 | 85 | 0.16 (0.13-0.19) | 53,894 | 1,495 | 2.77 (2.64-2.92) |
| **Hx of Ischemic stroke[2]** | | | | | | | | | | | | |
| **No** | | | | | | | | | | | | |

© 2022 Gaist D et al. *JAMA Network Open.*

| | | | | | | | | | | | | |
|---|---|---|---|---|---|---|---|---|---|---|---|---|
| ICH-cohort | 33,083 | 453 | 1.37 (1.25-1.50) | 33,083 | 175 | 0.53 (0.46-0.61) | 33,083 | 477 | 1.44 (1.32-1.58) | 32,857 | 1,313 | 4.00 (3.79-4.22) |
| | **Ischemic stroke** | | | **Myocardial infarct** | | | **Intracerebral hemorrhage** | | | **Major adverse cardiovascular events**[1] | | |
| ICH-cohort | 33,083 | 453 | 1.37 (1.25-1.50) | 33,083 | 175 | 0.53 (0.46-0.61) | 33,083 | 477 | 1.44 (1.32-1.58) | 32,857 | 1,313 | 4.00 (3.79-4.22) |
| Comparison cohort | 2,162,124 | 11,634 | 0.54 (0.53-0.55) | 2,162,124 | 10,340 | 0.48 (0.47-0.49) | 2,162,124 | 1,292 | 0.06 (0.06-0.06) | 2,157,045 | 28,285 | 1.31 (1.30-1.33) |
| **Diabetes**[2] | | | | | | | | | | | | |
| **Yes** | | | | | | | | | | | | |
| ICH-cohort | 3,916 | 69 | 1.76 (1.39-2.23) | 3,916 | 29 | 0.74 (0.51-1.07) | 3,916 | 54 | 1.38 (1.06-1.80) | 3,864 | 188 | 4.87 (4.22-5.61) |
| Comparison cohort | 192,541 | 1,488 | 0.77 (0.73-0.81) | 192,541 | 1,533 | 0.80 (0.76-0.84) | 192,541 | 134 | 0.07 (0.06-0.08) | 191,947 | 3,927 | 2.05 (1.98-2.11) |
| **Diabetes**[2] | | | | | | | | | | | | |
| **No** | | | | | | | | | | | | |
| ICH-cohort | 33,567 | 502 | 1.50 (1.37-1.63) | 33,567 | 165 | 0.49 (0.42-0.57) | 33,567 | 484 | 1.44 (1.32-1.58) | 33,344 | 1,359 | 4.08 (3.86-4.30) |
| Comparison cohort | 2,023,810 | 10,928 | 0.54 (0.53-0.55) | 2,023,810 | 9,136 | 0.45 (0.44-0.46) | 2,023,810 | 1,243 | 0.06 (0.06-0.06) | 2,018,992 | 25,853 | 1.28 (1.26-1.30) |
| **Chronic kidney failure**[2] | | | | | | | | | | | | |
| **Yes** | | | | | | | | | | | | |
| ICH-cohort | 453 | 14 | 3.09 | 453 | 7 | 1.54 | 453 | 12 | 2.65 | 452 | 41 | 9.08 |

© 2022 Gaist D et al. *JAMA Network Open.*

| | | | | | | | | | | | | |
|---|---|---|---|---|---|---|---|---|---|---|---|---|
| | | | (1.83-5.21) | | | (0.74-3.24) | | | (1.50-4.66) | | | (6.69-12.33) |
| | **Ischemic stroke** | | | **Myocardial infarct** | | | **Intracerebral hemorrhage** | | | **Major adverse cardiovascular events**[1] | | |
| Comparison cohort | 20,424 | 133 | 0.65 (0.55-0.77) | 20,424 | 198 | 0.97 (0.84-1.11) | 20,424 | 13 | 0.06 (0.04-0.11) | 20,360 | 497 | 2.44 (2.24-2.67) |
| **No** | | | | | | | | | | | | |
| ICH-cohort | 37,029 | 557 | 1.50 (1.38-1.63) | 37,029 | 187 | 0.51 (0.44-0.58) | 37,029 | 526 | 1.42 (1.30-1.55) | 36,756 | 1,506 | 4.10 (3.90-4.31) |
| Comparison cohort | 2,195,927 | 12,283 | 0.56 (0.55-0.57) | 2,195,927 | 10,471 | 0.48 (0.47-0.49) | 2,195,927 | 1,364 | 0.06 (0.06-0.07) | 2,190,579 | 29,283 | 1.34 (1.32-1.35) |
| **Chronic hepatic disease**[2] | | | | | | | | | | | | |
| **Yes** | | | | | | | | | | | | |
| ICH-cohort | 464 | 10 | 2.15 (1.16-4.00) | 464 | <5 | 0.86 (0.32-2.30) | 464 | 10 | 2.15 (1.16-4.00) | 464 | 30 | 6.46 (4.52-9.24) |
| Comparison cohort | 12,215 | 68 | 0.56 (0.44-0.71) | 12,215 | 63 | 0.52 (0.40-0.66) | 12,215 | 7 | 0.06 (0.03-0.12) | 12,188 | 196 | 1.61 (1.40-1.85) |
| **No** | | | | | | | | | | | | |
| ICH-cohort | 37,018 | 561 | 1.52 (1.40-1.65) | 37,018 | 190 | 0.51 (0.45-0.59) | 37,018 | 528 | 1.43 (1.31-1.55) | 36,744 | 1,517 | 4.13 (3.93-4.34) |
| Comparison cohort | 2,204,136 | 12,348 | 0.56 (0.55-0.57) | 2,204,136 | 10,606 | 0.48 (0.47-0.49) | 2,204,136 | 1,370 | 0.06 (0.06-0.07) | 2,198,752 | 29,584 | 1.35 (1.33-1.36) |
| **COPD**[2,3] | | | | | | | | | | | | |

© 2022 Gaist D et al. *JAMA Network Open.*

| | Ischemic stroke | | | Myocardial infarct | | | Intracerebral hemorrhage | | | Major adverse cardiovascular events[1] | | |
|---|---|---|---|---|---|---|---|---|---|---|---|---|
| **Yes** | | | | | | | | | | | | |
| ICH-cohort | 7,499 | 133 | 1.77 (1.50-2.10) | 7,499 | 48 | 0.64 (0.48-0.85) | 7,499 | 110 | 1.47 (1.22-1.77) | 7,447 | 353 | 4.74 (4.27-5.26) |
| Comparison cohort | 426,751 | 2,563 | 0.60 (0.58-0.62) | 426,751 | 2,495 | 0.58 (0.56-0.61) | 426,751 | 299 | 0.07 (0.06-0.08) | 425,459 | 6,751 | 1.59 (1.55-1.63) |
| **No** | | | | | | | | | | | | |
| ICH-cohort | 29,984 | 438 | 1.46 (1.33-1.60) | 29,984 | 146 | 0.49 (0.41-0.57) | 29,984 | 428 | 1.43 (1.30-1.57) | 29,761 | 1194 | 4.01 (3.79-4.25) |
| Comparison cohort | 1,789,600 | 9,853 | 0.55 (0.54-0.56) | 1,789,600 | 8,174 | 0.46 (0.45-0.47) | 1,789,600 | 1,078 | 0.06 (0.06-0.06) | 1,785,480 | 23,029 | 1.29 (1.27-1.31) |
| **High alcohol intake[2,4]** | | | | | | | | | | | | |
| **Yes** | | | | | | | | | | | | |
| ICH-cohort | 3,120 | 63 | 2.02 (1.58-2.58) | 3,120 | 11 | 0.35 (0.20-0.64) | 3,120 | 44 | 1.41 (1.05-1.90) | 3,109 | 148 | 4.76 (4.05-5.59) |
| Comparison cohort | 66,884 | 434 | 0.65 (0.59-0.71) | 66,884 | 313 | 0.47 (0.42-0.52) | 66,884 | 57 | 0.09 (0.07-0.11) | 66,690 | 1,049 | 1.57 (1.48-1.67) |
| **No** | | | | | | | | | | | | |
| ICH-cohort | 34,362 | 508 | 1.48 (1.36-1.61) | 34,362 | 183 | 0.53 (0.46-0.62) | 34,362 | 494 | 1.44 (1.32-1.57) | 34,098 | 1,399 | 4.10 (3.89-4.32) |
| Comparison cohort | 2,149,468 | 11,982 | 0.56 (0.55-0.57) | 2,149,468 | 10,356 | 0.48 (0.47-0.49) | 2,149,468 | 1,320 | 0.06 (0.06-0.06) | 2,144,249 | 28,731 | 1.34 (1.32-1.36) |

© 2022 Gaist D et al. *JAMA Network Open.*

[1]Defined as stroke [ischemic stroke, intracerebral hemorrhage, or ‘unspecified stroke’], myocardial infarction, systemic embolism, or vascular death
[2] Assessed at baseline based on data from previous 10 years.
[3]Chronic obstructive pulmonary disease.
[4]Disorder or drug use indicative of high alcohol intake.

© 2022 Gaist D et al. *JAMA Network Open.*

**eTable 3.** Hazard Ratio of Primary Outcomes and Select Secondary Outcomes in ICH Cohort, Overall and Stratified by Age, Sex, and Baseline Comorbidities (Comparison Cohort Used as Reference)

| | Event during follow-up | | | | | | | |
|---|---|---|---|---|---|---|---|---|
| | Ischemic stroke | | | Myocardial infarction | | | Intracerebral hemorrhage | |
| **Baseline characteristic /comorbidity** | HR[1] (95% CI) | aHR[2] (95% CI) | | HR[1] (95% CI) | aHR[2] (95% CI) | | HR[1] (95% CI) | aHR[2] (95% CI) |
| **All** | 2.79 (2.57-3.04) | 2.64 (2.43-2.88) | | 1.08 (0.93-1.24) | 1.12 (0.97-1.29) | | 23.7 (21.4-26.2) | 23.5 (21.1-26.1) |
| **Male sex** | 2.71 (2.43-3.04) | 2.48 (2.21-2.78) | | 0.98 (0.82-1.18) | 1.03 (0.86-1.24) | | 22.1 (19.2-25.4) | 22.1 (19.0-25.7) |
| **Female sex** | 2.89 (2.55-3.28) | 2.88 (2.53-3.27) | | 1.24 (0.98-1.56) | 1.29 (1.03-1.63) | | 25.6 (22.2-29.5) | 25.6 (22.0-29.7) |
| **Age 45-59 years** | 4.98 (4.05-6.11) | 3.21 (2.56-4.01) | | 1.66 (1.23-2.25) | 1.42 (1.04-1.95) | | 38.6 (28.5-52.2) | 31.2 (22.1-44.0) |
| **Age 60-74 years** | 3.03 (2.67-3.44) | 2.44 (2.14-2.78) | | 1.02 (0.81-1.28) | 0.98 (0.78-1.22) | | 30.5 (26.1-35.6) | 28.1 (23.8-33.1) |
| **Age 75-84 years** | 2.71 (2.33-3.14) | 2.46 (2.11-2.86) | | 1.00 (0.76-1.30) | 1.04 (0.79-1.36) | | 20.8 (17.6-24.7) | 18.2 (15.2-21.8) |
| **Age 85+ years** | 2.31 (1.71-3.10) | 1.94 (1.44-2.61) | | 1.32 (0.85-2.06) | 1.28 (0.82-2.00) | | 20.8 (14.7-29.5) | 18.2 (12.7-26.3) |
| **Atrial Fibrillation**[3] | | | | | | | | |
| Yes | 3.04 (2.51-3.68) | 2.99 (2.46-3.64) | | 1.16 (0.81-1.67) | 1.19 (0.82-1.72) | | 11.9 (8.8-16.9) | 10.8 (7.9-14.8) |
| No | 2.63 (2.39-2.88) | 2.56 (2.32-2.81) | | 1.04 (0.89-1.22) | 1.10 (0.94-1.28) | | 25.3 (22.8-28.1) | 26.2 (23.4-29.3) |
| **Hx of Ischemic stroke**[3] | | | | | | | | |
| Yes | 1.83 (1.51-2.23) | 2.01 (1.64-2.47) | | 0.71 (0.44-1.12) | 0.85 (0.53-1.37) | | 8.5 (6.1-11.8) | 9.7 (6.8-13.9) |
| No | 2.63 (2.39-2.89) | 2.68 (2.44-2.95) | | 1.11 (0.96-1.29) | 1.15 (0.99-1.33) | | 24.9 (22.4-27.7) | 25.4 (22.7-28.4) |
| **Diabetes**[3] | | | | | | | | |
| Yes | 2.31 (1.82-2.94) | 2.16 (1.68-2.76) | | 0.93 (0.64-1.34) | 1.03 (0.71-1.49) | | 19.9 (14.5-27.4) | 21.6 (15.4-30.4) |
| No | 2.85 (2.61-3.12) | 2.71 (2.47-2.97) | | 1.10 (0.94-1.28) | 1.13 (0.97-1.32) | | 24.1 (21.7-26.8) | 23.8 (21.3-26.6) |
| **Chronic kidney failure**[3] | | | | | | | | |

© 2022 Gaist D et al. *JAMA Network Open.*

| | Ischemic stroke | | | Myocardial infarction | | | Intracerebral hemorrhage | |
|---|---|---|---|---|---|---|---|---|
| Yes | 4.68 (2.70-8.14) | 3.62 (2.01-6.54) | | 1.56 (0.73-3.31) | 1.34 (0.61-2.93) | | 38.6 (17.5-85.0) | 77.84 (25.74-235.42) |
| No | 2.76 (2.54-3.01) | 2.61 (2.40-2.85) | | 1.06 (0.92-1.23) | 1.11 (0.96-1.28) | | 23.5 (21.2-26.0) | 23.2 (20.8-25.9) |
| **Chronic hepatic disease**[3] | | | | | | | | |
| Yes | 3.97 (2.03-7.74) | 4.17 (1.97-8.83) | | 1.57 (0.57-4.32) | 1.96 (0.67-5.72) | | 40.5 (15.3-107.7) | 582 (49.5-6841) |
| No | 2.78 (2.55-3.02) | 2.63 (2.41-2.86) | | 1.07 (0.93-1.23) | 1.12 (0.97-1.29) | | 23.5 (21.3-26.0) | 23.4 (21.0-26.0) |
| **COPD**[3, 4] | | | | | | | | |
| Yes | 3.00 (2.52-3.57) | 2.81 (2.35-3.36) | | 1.08 (0.81-1.44) | 1.14 (0.86-1.53) | | 21.6 (17.3-26.9) | 20.5 (16.2-26.0) |
| No | 2.73 (2.48-3.01) | 2.58 (2.34-2.85) | | 1.07 (0.91-1.26) | 1.11 (0.94-1.31) | | 24.2 (21.7-27.1) | 24.7 (21.9-27.8) |
| **High alcohol intake**[3, 4] | | | | | | | | |
| Yes | 3.22 (2.47-4.20) | 2.95 (2.23-3.90) | | 0.76 (0.42-1.38) | 0.78 (0.42-1.43) | | 16.0 (10.8-23.8) | 15.6 (10.2-24.0) |
| No | 2.72 (2.49-2.97) | 2.60 (2.37-2.84) | | 1.11 (0.96-1.28) | 1.15 (0.99-1.33) | | 24.0 (21.7-26.7) | 23.9 (21.5-26.7) |

© 2022 Gaist D et al. *JAMA Network Open.*

| | Event during follow-up | | | | | | | |
|---|---|---|---|---|---|---|---|---|
| | Major Vascular Event | | | Vascular death | | | Unspecified stroke | |
| **Baseline characteristic /comorbidity** | HR[1] (95% CI) | adjHR[2] (95% CI) | | HR[1] (95% CI) | aHR[2] (95% CI) | | HR[1] (95% CI) | aHR[2] (95% CI) |
| **All** | 3.15 (2.99-3.31) | 3.13 (2.97-3.30) | | 2.96 (2.58-3.41) | 3.20 (2.78-3.69) | | 3.64 (2.74-4.84) | 3.39 (2.53-4.53) |
| **Male sex** | 2.82 (2.63-3.02) | 2.77 (2.58-2.97) | | 2.85 (2.37-3.43) | 3.04 (2.52-3.67) | | 3.96 (2.73-5.74) | 3.50 (2.39-5.12) |
| **Female sex** | 3.62 (3.36-3.91) | 3.69 (3.42-3.99) | | 3.11 (2.51-3.84) | 3.43 (2.76-4.26) | | 3.27 (2.11-5.09) | 3.31 (2.12-5.19) |
| **Age 45-59 years** | 4.69 (4.12-5.34) | 3.38 (2.94-3.89) | | 8.80 (5.85-13.22) | 4.97 (3.18-7.79) | | 7.77 (3.49-17.28) | 4.09 (1.61-10.40) |
| **Age 60-74 years** | 3.45 (3.19-3.73) | 2.98 (2.75-3.23) | | 3.39 (2.65-4.35) | 2.84 (2.20-3.65) | | 5.23 (3.40-8.03) | 3.65 (2.33-5.71) |
| **Age 75-84 years** | 3.23 (2.96-3.53) | 3.10 (2.83-3.39) | | 3.46 (2.79-4.29) | 3.31 (2.66-4.11) | | 3.18 (1.95-5.19) | 2.95 (1.80-4.85) |
| **Age 85+ years** | 2.72 (2.29-3.24) | 2.43 (2.04-2.89) | | 2.61 (1.78-3.84) | 2.36 (1.60-3.47) | | 1.83 (0.68-4.94) | 1.77 (0.65-4.83) |
| **Atrial Fibrillation**[3] | | | | | | | | |
| Yes | 2.79 (2.46-3.18) | 2.85 (2.49-3.25) | | 1.73 (1.22-2.46) | 1.93 (1.35-2.75) | | 3.26 (1.70-6.27) | 3.32 (1.70-6.51) |
| No | 3.10 (2.93-3.27) | 3.16 (2.98-3.34) | | 3.10 (2.66-3.61) | 3.57 (3.06-4.17) | | 3.54 (2.58-4.85) | 3.35 (2.43-4.63) |
| **Hx of Ischemic stroke**[3] | | | | | | | | |
| Yes | 1.91 (1.67-2.20) | 2.24 (1.93; 2.59) | | 1.83 (1.28; 2.60) | 2.90 (1.99; 4.24) | | 1.84 (0.94-3.59) | 1.52 (0.75-3.11) |
| No | 3.12 (2.95; 3.30) | 3.21 (3.04- 3.40) | | 2.88 (2.47; 3.36) | 3.19 (2.73-3.72) | | 3.56 (2.60-4.88) | 3.62 (2.63-4.99) |
| **Diabetes**[3] | | | | | | | | |
| Yes | 2.41 (2.08-2.78) | 2.49 (2.14-2.89) | | 2.23 (1.54-3.23) | 2.65 (1.81-3.88) | | 3.85 (1.78-8.33) | 2.96 (1.32-6.62) |
| No | 3.26 (3.08-3.44) | 3.22 (3.05-3.41) | | 3.08 (2.65-3.58) | 3.29 (2.82-3.83) | | 3.60 (2.65-4.88) | 3.42 (2.51-4.68) |
| **Chronic kidney failure**[3] | | | | | | | | |
| Yes | 3.68 (2.68-5.07) | 3.27 (2.33-4.58) | | 2.05 (0.91-4.66) | 2.09 (0.90-4.89) | | 9.70 (2.12-44.45) | 12.29 (1.71-88.10) |
| No | 3.13 (2.97-3.30) | 3.10 (2.94-3.27) | | 2.99 (2.59-3.44) | 3.23 (2.80-3.73) | | 3.55 (2.66-4.74) | 3.30 (2.46-4.44) |

© 2022 Gaist D et al. *JAMA Network Open.*

| | Major Vascular Event | | | Vascular death | | | Unspecified stroke | |
|---|---|---|---|---|---|---|---|---|
| **Chronic hepatic disease**[3] | | | | | | | | |
| Yes | 4.01 (2.72-5.90) | 3.83 (2.50-5.88) | | 3.38 (1.53-7.45) | 2.67 (1.09-6.52) | | NA* | NA* |
| No | 3.13 (2.97-3.30) | 3.12 (2.96-3.29) | | 2.92 (2.53-3.37) | 3.19 (2.76-3.68) | | 3.68 (2.77-4.89) | 3.42 (2.56-4.57) |
| **COPD**[3,4] | | | | | | | | |
| Yes | 3.01 (2.71-3.36) | 3.03 (2.71-3.38) | | 2.74 (2.08-3.60) | 3.05 (2.30-4.03) | | 1.75 (0.78-3.95) | 1.59 (0.70-3.62) |
| No | 3.18 (3.00-3.38) | 3.15 (2.97-3.34) | | 3.04 (2.59-3.57) | 3.27 (2.77-3.85) | | 4.24 (3.13-5.75) | 3.91 (2.87-5.34) |
| **High alcohol intake**[3, 5] | | | | | | | | |
| Yes | 3.07 (2.59-3.65) | 2.90 (2.42-3.48) | | 2.78 (1.86-4.15) | 2.73 (1.79-4.16) | | 3.09 (0.91-10.51) | 2.54 (0.67-9.66) |
| No | 3.12 (2.96-3.29) | 3.13 (2.96-3.30) | | 2.87 (2.47-3.33) | 3.18 (2.74-3.70) | | 3.73 (2.78-5.00) | 3.45 (2.56-4.65) |

Abbreviation: NA: Not Applicable (too few events).
[1]Hazard ratio, unadjusted.
[2]Hazard ratio adjusted for sex, age (5-year bands), study period (4-5 year bands), baseline comorbidity [separate covariates for each of the following: hypertension, atrial fibrillation, IS, MI, systemic embolism, PAD, venous thromboembolism, diabetes, chronic kidney failure, chronic hepatic diseases, chronic obstructive pulmonary disorder, disorders indicative of high alcohol intake, congestive heart failure, and cancer] and baseline current use of drugs [separate covariates for each of the following drugs/drug classes: low-dose aspirin, clopidogrel, dipyridamole, vitamin K antagonist, direct oral anticoagulant, statins, thiazides and other non-loop diuretics, loop diuretics, beta blockers, calcium channel blockers, ACE-inhibitors and angiotensin II receptor blockers, nonsteroidal anti-inflammatory drugs, selective serotonin re-uptake inhibitors, and proton pump inhibitors].
[3]Assessed at baseline based on data from previous 10 years.
[4]Chronic obstructive pulmonary disease.
[5]Disorder indicative of high alcohol intake.

© 2022 Gaist D et al. *JAMA Network Open.*

**eTable 4.** Hazard Ratios for Primary Outcomes and Select Secondary Outcomes in ICH Cohort With Comparison Cohort as Reference and Including Estimates That Adjust for Death as Competing Event

| | ICH-cohort vs comparison cohort (reference) | | |
|---|---|---|---|
| **Primary outcomes and select secondary outcomes (in italics)** | HR[1] (95% CI) | aHR[2] (95% CI) | sdHR[3] (95% CI) |
| Ischemic stroke | 2.79 (2.57-3.04) | 2.64 (2.43-2.88) | 1.72 (1.58-1.88) |
| Intracerebral hemorrhage | 23.7 (21.4-26.2) | 23.5 (21.1-26.13) | 15.5 (13.9-17.2) |
| *'Unspecified stroke'* | 3.64 (2.74-4.84) | 3.39 (2.53-4.53) | 2.40 (1.79-3.20) |
| Myocardial infarction (MI) | 1.08 (0.93-1.24) | 1.12 (0.97-1.29) | 0.77 (0.67-0.89) |
| MACE[4] | 3.16 (3.01-3.33) | 3.14 (2.98-3.31) | 2.15 (2.04-2.27) |
| *Vascular death*[5] | 3.06 (2.64-3.55) | 3.30 (2.83-3.84) | 1.77 (1.54-2.04) |

[1]Hazard ratio, unadjusted.
[2]Hazard ratio adjusted for sex, age (5-year bands), study period (4-5 year bands), baseline comorbidity [separate covariates for each of the following: hypertension, atrial fibrillation, ischemic stroke, myocardial infarction, systemic embolism, peripheral arterial disease, venous thromboembolism, diabetes, chronic kidney failure, chronic hepatic diseases, chronic obstructive pulmonary disorder, disorders indicative of high alcohol intake, congestive heart failure, and cancer] and baseline current use of drugs [separate covariates for each of the following drugs/drug classes: low-dose aspirin, clopidogrel, dipyridamole, vitamin K antagonist, direct oral anticoagulant, statins, thiazides and other non-loop diuretics, loop diuretics, beta blockers, calcium channel blockers, ACE-inhibitors and angiotensin II receptor blockers, nonsteroidal anti-inflammatory drugs, selective serotonin re-uptake inhibitors, and proton pump inhibitors].
[3]Subdistibution hazard ratio adjusted for same covariates as above with all-cause-death as competing event.
[4]Major adverse cardiovascular event defined as stroke (ischemic stroke, intracerebral hemorrhage, or 'unspecified stroke'), myocardial infarction, systemic embolism, or vascular death.
[5]Death recorded as sudden cardiac death, or death within 30 days of any of following events: stroke (ischemic stroke, intracerebral hemorrhage, or unspecified stroke), myocardial infarction, systemic embolism, intracranial extra-axial hemorrhage, revascularization procedure, mesenteric ischemia, venous thromboembolism (i.e., deep vein thrombosis or pulmonary embolism), or extracranial hemorrhage.

© 2022 Gaist D et al. *JAMA Network Open.*

**eTable 5.** Incidence Rates of Primary and Secondary Study Outcomes Based on Individual Follow-up for Each Outcome With Censoring Only Contingent on Migration, Death, or End of Study Period (as Opposed to Main Analysis, Where First Occurrence of a Primary Outcome Was Also a Censoring Criterion)

| | ICH cohort | | | Comparison cohort | |
|---|---|---|---|---|---|
| **Outcome during follow-up** | No. of events / person-years | Event rate per 100 person-years (95% CI) | | No. of events / person-years | Event rate per 100 person-years (95% CI) |
| **Primary outcomes and select secondary outcomes (in italics)** | | | | | |
| Ischemic stroke (IS) | 595/39,279 | 1.51 (1.40-1.64) | | 12,855/2,256,688 | 0.57 (0.56-0.58) |
| Intracerebral hemorrhage (ICH) | 577/39,813 | 1.45 (1.34-1.57) | | 1,504/2,295,631 | 0.07 (0.06-0.07) |
| *'Unspecified stroke'* | 58/40,841 | 0.14 (0.11-0.18) | | 825/2,293,980 | 0.04 (0.03-0.04) |
| Myocardial infarct (MI) | 212/40,469 | 0.52 (0.46-0.60) | | 10,949/2,258,156 | 0.48 (0.48-0.49) |
| MACE[1] | 1,584/37,208 | 4.26 (4.05-4.47) | | 30,706/2,210,939 | 1.39 (1.37-1.40) |
| *Vascular death*[2] | 521/41,114 | 1.27 (1.16-1.38) | | 7,961/2,297,952 | 0.35 (0.34-0.35) |
| **Secondary outcomes** | | | | | |
| Mesenteric ischemia | <5/41,109 | 0.00 (0.00-0.02) | | 29/2,297,907 | 0.00 (0.00-0.00) |
| Systemic embolism | 32/41,046 | 0.08 (0.06-0.11) | | 859/2,295,486 | 0.04 (0.04-0.04) |
| Venous thromboembolism | 250/40,457 | 0.62 (0.55-0.70) | | 8,787/2,269,424 | 0.39 (0.38-0.40) |
| Intracranial extra-axial hemorrhage[3] | 213/40,411 | 0.53 (0.46-0.60) | | 2,473/2,291,304 | 0.11 (0.10-0.11) |
| Extracranial hemorrhage[4] | 681/39,181 | 1.74 (1.61-1.87) | | 22,723/2,219,206 | 1.02 (1.01-1.04) |
| Sudden cardiac death | 23/41,113 | 0.06 (0.04-0.08) | | 506/2,297,951 | 0.02 (0.02-0.02) |
| All-cause-death | 4,340/41,114 | 10.56 (10.25-1 0.87) | | 78,200/2,297,952 | 3.40 (3.38-3.43) |
| Major ischemic vascular event[5] | 1,346/36,940 | 3.64 (3.45-3.84) | | 45,570/2,131,314 | 2.14 (2.12-2.16) |

© 2022 Gaist D et al. *JAMA Network Open.*

| | ICH cohort | | | Comparison cohort | |
|---|---|---|---|---|---|
| | No. of events / person-years | Event rate per 100 person-years (95% CI) | | No. of events / person-years | Event rate per 100 person-years (95% CI) |
| Major hemorrhagic vascular event[6] | 1,385/37,369 | 3.71 (3.52-3.91) | | 26,199/2,211,136 | 1.18 (1.17-1.20) |
| Major arterial vascular event[7] | 1,137/37,518 | 3.03 (2.86-3.21) | | 38,450/2,155,634 | 1.78 (1.77-1.80) |
| Non-vascular death[9] | 3,819/41,114 | 9.29 (9.00-9.59) | | 70,239/2,297,952 | 3.06 (3.03-3.08) |

[1]Major adverse cardiovascular event defined as stroke (ischemic stroke, intracerebral hemorrhage, or 'unspecified stroke'), myocardial infarction, systemic embolism, or vascular death.
[2]Death recorded as sudden cardiac death, or death within 30 days of any of following events: stroke (ischemic stroke, intracerebral hemorrhage, or 'unspecified stroke'), myocardial infarction, systemic embolism, intracranial extraaxial hemorrhage, revascularization procedure, mesenteric ischemia, venous thromboembolism, or extracranial hemorrhage.
[3]Subdural hematoma, subarachnoid hemorrhage, or epidural hematoma.
[4]Gastrointestinal hemorrhage or other major extracranial hemorrhage (for full definition see text).
[5]Defined as ischemic stroke, 'unspecified stroke', myocardial infarction, systemic embolism, revascularization procedures, mesenteric ischemia, venous thromboembolism, or sudden cardiac death.
[6]Defined as gastrointestinal hemorrhage or other major extracranial hemorrhage (for full definition see text) and also including intracranial extraaxial hemorrhages.
[7]Defined as ischemic stroke, 'unspecified stroke', myocardial infarction, systemic embolism, revascularization procedures, mesenteric ischemia, or sudden cardiac death.
[8]Deaths that do not fulfill criteria for vascular death (see above).

© 2022 Gaist D et al. *JAMA Network Open.*

**eTable 6.** Nested Case-Control Analysis of the Risk of Ischemic Stroke During Follow-up Associated With Having Had an Intracerebral Hemorrhage at Baseline

| | No. (%) cases | No. (%) controls | OR[1] (95% CI) | Adj.OR[2] (95% CI) |
|---|---|---|---|---|
| **All** | 12,987 | 216,885 | | |
| No previous ICH[3] | 12,416 (95.6) | 213,060 (98.2) | 1 (reference) | 1 (reference) |
| Previous ICH[4] | 571 (4.4) | 3,825 (1.8) | 2.63 (2.40-2.87) | 2.00 (1.82-2.20) |
| **Sex** | | | | |
| Male | 7,200 | 120,247 | | |
| No previous ICH[3] | 6,881 (95.6) | 118,086 (98.2) | 1 (reference) | 1 (reference) |
| Previous ICH[4] | 319 (4.4) | 2,161 (1.8) | 2.55 (2.26-2.88) | 1.89 (1.66-2.14) |
| Female | 5,787 | 96,638 | | |
| No previous ICH[3] | 5,535 (95.6) | 94,974 (98.3) | 1 (reference) | 1 (reference) |
| Previous ICH[4] | 252 (4.4) | 1,664 (1.7) | 2.73 (2.38-3.13) | 2.17 (1.88-2.51) |
| **Age on index date** | | | | |
| <75 years | 6,168 | 119,085 | | |
| No previous ICH[3] | 5,820 (94.4) | 116,899 (98.2) | 1 (reference) | 1 (reference) |
| Previous ICH[4] | 348 (5.6) | 2,186 (1.8) | 3.36 (2.98-3.78) | 2.25 (1.98-2.56) |
| ≥75 years | 6,819 | 97,800 | | |
| No previous ICH[3] | 6,596 (96.7) | 96,161 (98.3) | 1 (reference) | 1 (reference) |
| Previous ICH[4] | 223 (3.3) | 1,639 (1.7) | 2.00 (1.73-2.31) | 1.62 (1.39-1.89) |
| | **No. (%) cases** | **No. (%) controls** | **OR[5] (95% CI)** | **Adj.OR[6] (95% CI)** |
| **Atrial fibrillation**[7] | | | | |
| Yes | 3,106 | 24,878 | | |
| No previous ICH[3] | 2,952 (95.0) | 24,225 (97.4) | 1 (reference) | 1 (reference) |
| Previous ICH[4] | 154 (5.0) | 653 (2.6) | 2.11 (1.76-2.54) | 1.61 (1.33-1.95) |
| No | 9,881 | 192,007 | | |
| No previous ICH[3] | 9,464 (95.8) | 188,835 (98.3) | 1 (reference) | 1 (reference) |
| Previous ICH[4] | 417 (4.2) | 3,172 (1.7) | 2.73 (2.46-3.03) | 2.30 (2.07-2.57) |
| **Diabetes**[7] | | | | |
| Yes | 2,198 | 28,224 | | |
| No previous ICH[3] | 2,101 (95.6) | 27,660 (98.0) | 1 (reference) | 1 (reference) |

© 2022 Gaist D et al. *JAMA Network Open.*

| | No. (%) cases | No. (%) controls | OR[5] (95% CI) | Adj.OR[6] (95% CI) |
|---|---|---|---|---|
| Previous ICH[4] | 97 (4.4) | 564 (2.0) | 2.31 (1.85-2.88) | 1.81 (1.44-2.28) |
| No | 10,789 | 188,661 | | |
| No previous ICH[3] | 10,315 (95.6) | 185,400 (98.3) | 1 (reference) | 1 (reference) |
| Previous ICH[4] | 474 (4.4) | 3,261 (1.7) | 2.74 (2.48-3.03) | 2.16 (1.94-2.39) |
| **History of major ischemic vascular event**[8] | | | | |
| Yes | 2,091 | 24,811 | | |
| No previous ICH[3] | 1,861 (89.0) | 23,561 (95.0) | 1 (reference) | 1 (reference) |
| Previous ICH[4] | 230 (11.0) | 1,250 (5.0) | 2.08 (1.79-2.42) | 1.83 (1.55-2.15) |
| No | 10,896 | 192,074 | | |
| No previous ICH[3] | 10,555 (96.9) | 189,499 (98.7) | 1 (reference) | 1 (reference) |
| Previous ICH[4] | 341 (3.1) | 2,575 (1.3) | 2.56 (2.28-2.88) | 2.12 (1.88-2.39) |
| **History of ischemic stroke before baseline**[9] | | | | |
| Yes | 722 | 5,588 | | |
| No previous ICH[3] | 616 (85.3) | 5,141 (92.0) | 1 (reference) | 1 (reference) |
| Previous ICH[4] | 106 (14.7) | 447 (8.0) | 1.54 (1.22-1.96) | 1.57 (1.22-2.02) |
| No | 12,265 | 211,297 | | |
| No previous ICH[3] | 11,800 (96.2) | 207,919 (98.4) | 1 (reference) | 1 (reference) |
| Previous ICH[4] | 465 (3.8) | 3,378 (1.6) | 2.55 (2.30-2.81) | 2.11 (1.90-2.34) |
| **History of major ischemic vascular event other than ischemic stroke**[10] | | | | |
| Yes | 1,245 | 17,561 | | |
| No previous ICH[3] | 1,166 (93.7) | 17,153 (97.7) | 1 (reference) | 1 (reference) |
| Previous ICH[4] | 79 (6.3) | 408 (2.3) | 2.87 (2.23-3.69) | 2.31 (1.76-3.01) |
| No | 11,742 | 199,324 | | |
| No previous ICH[3] | 11,250 (95.8) | 195,907 (98.3) | 1 (reference) | 1 (reference) |
| Previous ICH[4] | 492 (4.2) | 3,417 (1.7) | 2.63 (2.39-2.90) | 2.06 (1.86-2.28) |

[1]Adjusted for age, sex, and index date (by design).

© 2022 Gaist D et al. *JAMA Network Open.*

[2]Adjusted for sex, age, and index date (by design), comorbidity [separate covariates for each of the following: hypertension, atrial fibrillation, ischemic stroke, myocardial infarction, systemic embolism, peripheral arterial disease, venous thromboembolism, diabetes, chronic kidney failure, chronic hepatic diseases, chronic obstructive pulmonary disorder, disorders indicative of high alcohol intake, congestive heart failure, and cancer] and current use of drugs [separate covariates for each of the following drugs/drug classes: low-dose aspirin, clopidogrel, dipyridamole, vitamin K antagonist, direct oral anticoagulant, statins, thiazides and other non-loop diuretics, loop diuretics, beta blockers, calcium channel blockers, ACE-inhibitors and angiotensin II receptor blockers, nonsteroidal anti-inflammatory drugs, selective serotonin re-uptake inhibitors, and proton pump inhibitors]. All covariates were determined on index-date and based on all available data, i.e., from 10 years before baseline and up to index-date.
[3]No history of intracerebral hemorrhage at baseline (i.e., stems from comparison-cohort).
[4]First-ever intracerebral hemorrhage at baseline (i.e., stems from ICH-cohort).
[5]Adjusted for age (5-year bands), sex, and index date year (3-year bands).
[6]Adjusted for age (5-year bands), sex, index date year (3-year bands), duration of follow-up (4-year bands), comorbidity [separate covariates for each of the following: hypertension, atrial fibrillation, ischemic stroke, myocardial infarction, systemic embolism, peripheral arterial disease, venous thromboembolism, diabetes, chronic kidney failure, chronic hepatic diseases, chronic obstructive pulmonary disorder, disorders indicative of high alcohol intake, congestive heart failure, and cancer] and current use of drugs [separate covariates for each of the following drugs/drug classes: low-dose aspirin, clopidogrel, dipyridamole, vitamin K antagonist, direct oral anticoagulant, statins, thiazides and other non-loop diuretics, loop diuretics, beta blockers, calcium channel blockers, ACE-inhibitors and angiotensin II receptor blockers, nonsteroidal anti-inflammatory drugs, selective serotonin re-uptake inhibitors, and proton pump inhibitors]. All covariates were determined on index-date and based on all available data, i.e., from 10 years before baseline and up to index-date.
[7]At baseline or during follow-up.
[8]Defined as ischemic stroke or 'unspecified stroke' (only if recorded before inclusion into baseline), myocardial infarction, systemic embolism, revascularization procedures, mesenteric ischemia, venous thromboembolism, or sudden cardiac death.
[9]Only ischemic strokes recorded before baseline (i.e., before inclusion into cohorts) included.
[10]Defined as in footnote 8 but excluding ischemic stroke or 'unspecified stroke'.

© 2022 Gaist D et al. *JAMA Network Open.*

**eTable 7.** Nested Case-Control Analysis of the Risk of Intracerebral Hemorrhage During Follow-up Associated With Having Had an Intracerebral Hemorrhage at Baseline

| | No. (%) cases | No. (%) controls | OR[1] (95% CI) | Adj.OR[2] (95% CI) |
|---|---|---|---|---|
| **All** | 1,915 | 64,955 | | |
| No previous ICH[3] | 1,377 (71.9) | 63,958 (98.5) | 1 (reference) | 1 (reference) |
| Previous ICH[4] | 538 (28.1) | 997 (1.5) | 26.65 (23.56-30.16) | 25.59 (22.30-29.36) |
| **Sex** | | | | |
| Male | 973 | 33,185 | | |
| No previous ICH[3] | 707 (72.7) | 32,689 (98.5) | 1 (reference) | 1 (reference) |
| Previous ICH[4] | 266 (27.3) | 496 (1.5) | 26.27 (22.07-31.29) | 26.87 (22.08-32.71) |
| Female | 942 | 31,770 | | |
| No previous ICH[3] | 670 (71.1) | 31,269 (98.4) | 1 (reference) | 1 (reference) |
| Previous ICH[4] | 272 (28.9) | 501 (1.6) | 27.04 (22.70-32.21) | 24.89 (20.49-30.23) |
| **Age on index date** | | | | |
| <75 years | 900 | 32,087 | | |
| No previous ICH[3] | 578 (64.2) | 31,570 (98.4) | 1 (reference) | 1 (reference) |
| Previous ICH[4] | 322 (35.8) | 517 (1.6) | 37.98 (31.78-45.38) | 35.74 (29.09-43.91) |
| ≥75 years | 1,015 | 32,868 | | |
| No previous ICH[3] | 799 (78.7) | 32,388 (98.5) | 1 (reference) | 1 (reference) |
| Previous ICH[4] | 216 (21.3) | 480 (1.5) | 18.82 (15.55-22.77) | 19.27 (15.62-23.78) |
| | **No. (%) cases** | **No. (%) controls** | **OR[5] (95% CI)** | **Adj.OR[6] (95% CI)** |
| **Atrial fibrillation**[7] | | | | |
| Yes | 416 | 7,683 | | |
| No previous ICH[3] | 334 (80.3) | 7,510 (97.7) | 1 (reference) | 1 (reference) |
| Previous ICH[4] | 82 (19.7) | 173 (2.3) | 10.97 (8.21-14.65) | 13.38 (9.60-18.65) |
| No | 1,499 | 57,272 | | |
| No previous ICH[3] | 1,043 (69.6) | 56,448 (98.6) | 1 (reference) | 1 (reference) |
| Previous ICH[4] | 456 (30.4) | 824 (1.4) | 30.67 (26.91-34.96) | 30.76 (26.54-35.66) |
| **Diabetes**[7] | | | | |
| Yes | 246 | 8,351 | | |
| No previous ICH[3] | 178 (72.4) | 8,212 (98.3) | 1 (reference) | 1 (reference) |
| Previous ICH[4] | 68 (27.6) | 139 (1.7) | 23.22 (16.68-32.34) | 23.27 (15.91-34.03) |

© 2022 Gaist D et al. *JAMA Network Open.*

| | No. (%) cases | No. (%) controls | OR[5] (95% CI) | Adj.OR[6] (95% CI) |
|---|---|---|---|---|
| No | 1,669 | 56,604 | | |
| No previous ICH[3] | 1,199 (71.8) | 55,746 (98.5) | 1 (reference) | 1 (reference) |
| Previous ICH[4] | 470 (28.2) | 858 (1.5) | 26.16 (23.03-29.71) | 27.47 (23.78-31.74) |
| **History of major ischemic vascular event**[8] | | | | |
| Yes | 361 | 6,998 | | |
| No previous ICH[3] | 181 (50.1) | 6,671 (95.3) | 1 (reference) | 1 (reference) |
| Previous ICH[4] | 180 (49.9) | 327 (4.7) | 18.21 (14.26-23.26) | 17.81 (13.42-23.62) |
| No | 1,554 | 57,957 | | |
| No previous ICH[3] | 1,196 (77.0) | 57,287 (98.8) | 1 (reference) | 1 (reference) |
| Previous ICH[4] | 358 (23.0) | 670 (1.2) | 27.03 (23.44-31.18) | 29.52 (25.19-34.60) |
| **History of ischemic stroke before baseline**[9] | | | | |
| Yes | 128 | 1,581 | | |
| No previous ICH[3] | 71 (55.5) | 1,462 (92.5) | 1 (reference) | 1 (reference) |
| Previous ICH[4] | 57 (44.5) | 119 (7.5) | 8.33 (5.49-12.64) | 9.93 (6.03-16.35) |
| No | 1,787 | 63,374 | | |
| No previous ICH[3] | 1,306 (73.1) | 62,496 (98.6) | 1 (reference) | 1 (reference) |
| Previous ICH[4] | 481 (26.9) | 878 (1.4) | 27.12 (23.93-30.74) | 28.55 (24.82-32.84) |
| **History of major ischemic vascular event other than ischemic stroke**[10] | | | | |
| Yes | 142 | 4,891 | | |
| No previous ICH[3] | 89 (62.7) | 4,802 (98.2) | 1 (reference) | 1 (reference) |
| Previous ICH[4] | 53 (37.3) | 89 (1.8) | 31.11 (20.67-46.82) | 37.24 (22.89-60.60) |
| No | 1,773 | 60,064 | | |
| No previous ICH[3] | 1,288 (72.6) | 59,156 (98.5) | 1 (reference) | 1 (reference) |
| Previous ICH[4] | 485 (27.4) | 908 (1.5) | 25.35 (22.38-28.71) | 25.71 (22.34-29.60) |

[1]Adjusted for age, sex, and index date (by design).
[2]Adjusted for sex, age, and index date (by design), comorbidity [separate covariates for each of the following: hypertension, atrial fibrillation, ischemic stroke, myocardial infarction, systemic embolism, peripheral arterial disease, venous thromboembolism, diabetes, chronic kidney failure, chronic hepatic diseases, chronic obstructive pulmonary disorder, disorders indicative of high alcohol intake, congestive heart failure, and cancer] and current use of drugs [separate covariates for each of the following drugs/drug classes: low-dose aspirin, clopidogrel, dipyridamole, vitamin K antagonist, direct oral anticoagulant, statins, thiazides and other non-loop diuretics,

© 2022 Gaist D et al. *JAMA Network Open.*

loop diuretics, beta blockers, calcium channel blockers, ACE-inhibitors and angiotensin II receptor blockers, nonsteroidal anti-inflammatory drugs, selective serotonin re-uptake inhibitors, and proton pump inhibitors]. All covariates were determined on index-date and based on all available data, i.e., from 10 years before baseline and up to index-date.
[3]No history of intracerebral hemorrhage at baseline (i.e., stems from comparison-cohort).
[4]First-ever intracerebral hemorrhage at baseline (i.e., stems from ICH-cohort).
[5]Adjusted for age (5-year bands), sex, and index date year (3-year bands).
[6]Adjusted for age (5-year bands), sex, index date year (3-year bands), duration of follow-up (4-year bands), comorbidity [separate covariates for each of the following: hypertension, atrial fibrillation, ischemic stroke, myocardial infarction, systemic embolism, peripheral arterial disease, venous thromboembolism, diabetes, chronic kidney failure, chronic hepatic diseases, chronic obstructive pulmonary disorder, disorders indicative of high alcohol intake, congestive heart failure, and cancer] and current use of drugs [separate covariates for each of the following drugs/drug classes: low-dose aspirin, clopidogrel, dipyridamole, vitamin K antagonist, direct oral anticoagulant, statins, thiazides and other non-loop diuretics, loop diuretics, beta blockers, calcium channel blockers, ACE-inhibitors and angiotensin II receptor blockers, nonsteroidal anti-inflammatory drugs, selective serotonin re-uptake inhibitors, and proton pump inhibitors]. All covariates were determined on index-date and based on all available data, i.e., from 10 years before baseline and up to index-date.
[7]At baseline or during follow-up.
[8]Defined as ischemic stroke or 'unspecified stroke' (only if recorded before inclusion into baseline), myocardial infarction, systemic embolism, revascularization procedures, mesenteric ischemia, venous thromboembolism, or sudden cardiac death.
[9]Only ischemic strokes recorded before baseline (i.e., before inclusion into cohorts) included.
[10]Defined as in footnote 8 but excluding ischemic stroke or 'unspecified stroke'.

© 2022 Gaist D et al. *JAMA Network Open.*

**eTable 8.** Nested Case-Control Analysis of the Risk of Myocardial Infarction During Follow-up Associated With Having Had an Intracerebral Hemorrhage at Baseline

| | No. (%) cases | No. (%) controls | OR[1] (95% CI) | Adj.OR[2] (95% CI) |
|---|---|---|---|---|
| **All** | 10,863 | 203,928 | | |
| No previous ICH[3] | 10,669 (98.2) | 200,237 (98.2) | 1 (reference) | 1 (reference) |
| Previous ICH[4] | 194 (1.8) | 3,691 (1.8) | 0.98 (0.85-1.14) | 0.89 (0.76-1.03) |
| **Sex** | | | | |
| Male | 7,052 | 121,590 | | |
| No previous ICH[3] | 6,933 (98.3) | 119,296 (98.1) | 1 (reference) | 1 (reference) |
| Previous ICH[4] | 119 (1.7) | 2,294 (1.9) | 0.89 (0.74-1.08) | 0.83 (0.68-1.00) |
| Female | 3,811 | 82,338 | | |
| No previous ICH[3] | 3,736 (98.0) | 80,941 (98.3) | 1 (reference) | 1 (reference) |
| Previous ICH[4] | 75 (2.0) | 1,397 (1.7) | 1.16 (0.92-1.47) | 1.01 (0.79-1.29) |
| **Age on index date** | | | | |
| <75 years | 5,735 | 115,947 | | |
| No previous ICH[3] | 5,615 (97.9) | 113,724 (98.1) | 1 (reference) | 1 (reference) |
| Previous ICH[4] | 120 (2.1) | 2,223 (1.9) | 1.09 (0.91-1.32) | 0.92 (0.76-1.12) |
| ≥75 years | 5,128 | 87,981 | | |
| No previous ICH[3] | 5,054 (98.6) | 86,513 (98.3) | 1 (reference) | 1 (reference) |
| Previous ICH[4] | 74 (1.4) | 1,468 (1.7) | 0.84 (0.66-1.07) | 0.86 (0.67-1.10) |
| | **No. (%) cases** | **No. (%) controls** | **OR[5] (95% CI)** | **Adj.OR[6] (95% CI)** |
| **Atrial fibrillation**[7] | | | | |
| Yes | 1,898 | 22,510 | | |
| No previous ICH[3] | 1,847 (97.3) | 21,859 (97.1) | 1 (reference) | 1 (reference) |
| Previous ICH[4] | 51 (2.7) | 651 (2.9) | 0.99 (0.74-1.32) | 0.88 (0.65-1.18) |
| No | 8,965 | 181,418 | | |
| No previous ICH[3] | 8,822 (98.4) | 178,378 (98.3) | 1 (reference) | 1 (reference) |
| Previous ICH[4] | 143 (1.6) | 3,040 (1.7) | 0.97 (0.82-1.14) | 0.92 (0.77-1.09) |
| **Diabetes**[7] | | | | |
| Yes | 2,081 | 25,971 | | |
| No previous ICH[3] | 2,039 (98.0) | 25,400 (97.8) | 1 (reference) | 1 (reference) |

| | No. (%) cases | No. (%) controls | OR[5] (95% CI) | Adj.OR[6] (95% CI) |
|---|---|---|---|---|
| Previous ICH[4] | 42 (2.0) | 571 (2.2) | 0.93 (0.68-1.28) | 0.92 (0.66-1.27) |
| No | 8,782 | 177,957 | | |
| No previous ICH[3] | 8,630 (98.3) | 174,837 (98.2) | 1 (reference) | 1 (reference) |
| Previous ICH[4] | 152 (1.7) | 3,120 (1.8) | 1.01 (0.85-1.19) | 0.92 (0.78-1.09) |
| **History of major ischemic vascular event[8]** | | | | |
| Yes | 2,031 | 23,296 | | |
| No previous ICH[3] | 1,940 (95.5) | 22,075 (94.8) | 1 (reference) | 1 (reference) |
| Previous ICH[4] | 91 (4.5) | 1,221 (5.2) | 0.74 (0.59-0.92) | 1.06 (0.84-1.33) |
| No | 8,832 | 180,632 | | |
| No previous ICH[3] | 8,729 (98.8) | 178,162 (98.6) | 1 (reference) | 1 (reference) |
| Previous ICH[4] | 103 (1.2) | 2,470 (1.4) | 0.89 (0.73-1.09) | 0.78 (0.63-0.96) |
| **History of ischemic stroke before baseline[9]** | | | | |
| Yes | 251 | 5,377 | | |
| No previous ICH[3] | 234 (93.2) | 4,937 (91.8) | 1 (reference) | 1 (reference) |
| Previous ICH[4] | 17 (6.8) | 440 (8.2) | 0.78 (0.46-1.29) | 0.80 (0.47-1.36) |
| No | 10,612 | 198,551 | | |
| No previous ICH[3] | 10,435 (98.3) | 195,300 (98.4) | 1 (reference) | 1 (reference) |
| Previous ICH[4] | 177 (1.7) | 3,251 (1.6) | 1.04 (0.89-1.21) | 0.93 (0.80-1.09) |
| **History of major ischemic vascular event other than ischemic stroke[10]** | | | | |
| Yes | 1,725 | 16,249 | | |
| No previous ICH[3] | 1,677 (97.2) | 15,865 (97.6) | 1 (reference) | 1 (reference) |
| Previous ICH[4] | 48 (2.8) | 384 (2.4) | 1.11 (0.81-1.51) | 1.17 (0.85-1.62) |
| No | 9,138 | 187,679 | | |
| No previous ICH[3] | 8,992 (98.4) | 184,372 (98.2) | 1 (reference) | 1 (reference) |
| Previous ICH[4] | 146 (1.6) | 3,307 (1.8) | 0.93 (0.78-1.10) | 0.84 (0.71-1.00) |

[1]Adjusted for age, sex, and index date (by design).
[2]Adjusted for sex, age, and index date (by design), comorbidity [separate covariates for each of the following: hypertension, atrial fibrillation, ischemic stroke, myocardial infarction, systemic embolism, peripheral arterial disease, venous thromboembolism, diabetes, chronic kidney failure, chronic hepatic diseases, chronic obstructive pulmonary disorder, disorders indicative of high alcohol intake,

© 2022 Gaist D et al. *JAMA Network Open.*

congestive heart failure, and cancer] and current use of drugs [separate covariates for each of the following drugs/drug classes: low-dose aspirin, clopidogrel, dipyridamole, vitamin K antagonist, direct oral anticoagulant, statins, thiazides and other non-loop diuretics, loop diuretics, beta blockers, calcium channel blockers, ACE-inhibitors and angiotensin II receptor blockers, nonsteroidal anti-inflammatory drugs, selective serotonin re-uptake inhibitors, and proton pump inhibitors]. All covariates were determined on index-date and based on all available data, i.e., from 10 years before baseline and up to index-date.
[3]No history of intracerebral hemorrhage at baseline (i.e., stems from comparison-cohort).
[4]First-ever intracerebral hemorrhage at baseline (i.e., stems from ICH-cohort).
[5]Adjusted for age (5-year bands), sex, and index date year (3-year bands).
[6]Adjusted for age (5-year bands), sex, index date year (3-year bands), duration of follow-up (4-year bands), comorbidity [separate covariates for each of the following: hypertension, atrial fibrillation, ischemic stroke, myocardial infarction, systemic embolism, peripheral arterial disease, venous thromboembolism, diabetes, chronic kidney failure, chronic hepatic diseases, chronic obstructive pulmonary disorder, disorders indicative of high alcohol intake, congestive heart failure, and cancer] and current use of drugs [separate covariates for each of the following drugs/drug classes: low-dose aspirin, clopidogrel, dipyridamole, vitamin K antagonist, direct oral anticoagulant, statins, thiazides and other non-loop diuretics, loop diuretics, beta blockers, calcium channel blockers, ACE-inhibitors and angiotensin II receptor blockers, nonsteroidal anti-inflammatory drugs, selective serotonin re-uptake inhibitors, and proton pump inhibitors]. All covariates were determined on index-date and based on all available data, i.e., from 10 years before baseline and up to index-date.
[7]At baseline or during follow-up.
[8]Defined as ischemic stroke or ‘unspecified stroke’ (only if recorded before inclusion into baseline), myocardial infarction, systemic embolism, revascularization procedures, mesenteric ischemia, venous thromboembolism, or sudden cardiac death.
[9]Only ischemic strokes recorded before baseline (i.e., before inclusion into cohorts) included.
[10]Defined as in footnote 8 but excluding ischemic stroke or ‘unspecified stroke’.

© 2022 Gaist D et al. *JAMA Network Open.*

**eTable 9.** Nested Case-Control Analysis of the Risk of Major Adverse Cardiovascular Event (Stroke, Myocardial Infarction, Systemic Embolism, or Vascular Death) During Follow-up Associated With Having Had an Intracerebral Hemorrhage at Baseline

| | No. (%) cases | No. (%) controls | OR[1] (95% CI) | Adj.OR[2] (95% CI) |
|---|---|---|---|---|
| **All** | 31,327 | 269,891 | | |
| No previous ICH[3] | 29,780 (95.1) | 264,945 (98.2) | 1 (reference) | 1 (reference) |
| Previous ICH[4] | 1,547 (4.9) | 4,946 (1.8) | 2.89 (2.72-3.07) | 2.66 (2.49-2.84) |
| **Sex** | | | | |
| Male | 18,349 | 149,022 | | |
| No previous ICH[3] | 17,505 (95.4) | 146,191 (98.1) | 1 (reference) | 1 (reference) |
| Previous ICH[4] | 844 (4.6) | 2,831 (1.9) | 2.57 (2.37-2.79) | 2.29 (2.10-2.50) |
| Female | 12,978 | 120,869 | | |
| No previous ICH[3] | 12,275 (94.6) | 118,754 (98.3) | 1 (reference) | 1 (reference) |
| Previous ICH[4] | 703 (5.4) | 2,115 (1.7) | 3.38 (3.09-3.70) | 3.29 (2.98-3.64) |
| **Age on index date** | | | | |
| <75 years | 14,727 | 159,131 | | |
| No previous ICH[3] | 13,825 (93.9) | 156,180 (98.1) | 1 (reference) | 1 (reference) |
| Previous ICH[4] | 902 (6.1) | 2,951 (1.9) | 3.78 (3.49-4.10) | 3.12 (2.85-3.41) |
| ≥75 years | 16,600 | 110,760 | | |
| No previous ICH[3] | 15,955 (96.1) | 108,765 (98.2) | 1 (reference) | 1 (reference) |
| Previous ICH[4] | 645 (3.9) | 1,995 (1.8) | 2.19 (1.99-2.42) | 2.24 (2.02-2.49) |
| | **No. (%) cases** | **No. (%) controls** | **OR[5] (95% CI)** | **Adj.OR[6] (95% CI)** |
| **Atrial fibrillation[7]** | | | | |
| Yes | 6,971 | 26,154 | | |
| No previous ICH[3] | 6,618 (94.9) | 25,448 (97.3) | 1 (reference) | 1 (reference) |
| Previous ICH[4] | 353 (5.1) | 706 (2.7) | 2.15 (1.88-2.46) | 1.98 (1.72-2.28) |
| No | 24,265 | 210,517 | | |
| No previous ICH[3] | 23,079 (95.1) | 207,202 (98.4) | 1 (reference) | 1 (reference) |
| Previous ICH[4] | 1,186 (4.9) | 3,315 (1.6) | 3.39 (3.16-3.63) | 3.09 (2.88-3.32) |
| **Diabetes[7]** | | | | |
| Yes | 5,627 | 30,435 | | |

© 2022 Gaist D et al. *JAMA Network Open.*

| | No. (%) cases | No. (%) controls | OR[5] (95% CI) | Adj.OR[6] (95% CI) |
|---|---|---|---|---|
| No previous ICH[3] | 5,373 (95.5) | 29,820 (98.0) | 1 (reference) | 1 (reference) |
| Previous ICH[4] | 254 (4.5) | 615 (2.0) | 2.44 (2.10-2.84) | 2.31 (1.97-2.71) |
| No | 25,609 | 206,236 | | |
| No previous ICH[3] | 24,324 (95.0) | 202,830 (98.3) | 1 (reference) | 1 (reference) |
| Previous ICH[4] | 1,285 (5.0) | 3,406 (1.7) | 3.34 (3.12-3.57) | 2.93 (2.73-3.14) |
| **History of major ischemic vascular event**[8] | | | | |
| Yes | 5,622 | 26,243 | | |
| No previous ICH[3] | 5,024 (89.4) | 24,940 (95.0) | 1 (reference) | 1 (reference) |
| Previous ICH[4] | 598 (10.6) | 1,303 (5.0) | 2.03 (1.83-2.25) | 2.22 (1.98-2.49) |
| No | 25,614 | 210,428 | | |
| No previous ICH[3] | 24,673 (96.3) | 207,710 (98.7) | 1 (reference) | 1 (reference) |
| Previous ICH[4] | 941 (3.7) | 2,718 (1.3) | 3.21 (2.97-3.47) | 2.99 (2.76-3.24) |
| **History of ischemic stroke before baseline**[9] | | | | |
| Yes | 1,348 | 6,039 | | |
| No previous ICH[3] | 1,137 (84.3) | 5,591 (92.6) | 1 (reference) | 1 (reference) |
| Previous ICH[4] | 211 (15.7) | 448 (7.4) | 1.98 (1.65-2.38) | 1.95 (1.60-2.37) |
| **History of ischemic stroke before baseline**[9] | | | | |
| No | 29,888 | 230,632 | | |
| No previous ICH[3] | 28,560 (95.6) | 227,059 (98.5) | 1 (reference) | 1 (reference) |
| Previous ICH[4] | 1,328 (4.4) | 3,573 (1.5) | 3.15 (2.95-3.36) | 2.86 (2.67-3.06) |
| **History of major ischemic vascular event other than ischemic stroke**[10] | | | | |
| Yes | 3,916 | 18,389 | | |
| No previous ICH[3] | 3,696 (94.4) | 17,978 (97.8) | 1 (reference) | 1 (reference) |
| Previous ICH[4] | 220 (5.6) | 411 (2.2) | 2.58 (2.17-3.06) | 2.30 (1.91-2.76) |
| No | 27,320 | 218,282 | | |

© 2022 Gaist D et al. *JAMA Network Open.*

| | No. (%) cases | No. (%) controls | OR[5] (95% CI) | Adj.OR[6] (95% CI) |
|---|---|---|---|---|
| No previous ICH[3] | 26,001 (95.2) | 214,672 (98.3) | 1 (reference) | 1 (reference) |
| Previous ICH[4] | 1,319 (4.8) | 3,610 (1.7) | 3.23 (3.02-3.45) | 2.90 (2.70-3.11) |

[1]Adjusted for age, sex, and index date (by design).
[2]Adjusted for sex, age, and index date (by design), comorbidity [separate covariates for each of the following: hypertension, atrial fibrillation, ischemic stroke, myocardial infarction, systemic embolism, peripheral arterial disease, venous thromboembolism, diabetes, chronic kidney failure, chronic hepatic diseases, chronic obstructive pulmonary disorder, disorders indicative of high alcohol intake, congestive heart failure, and cancer] and current use of drugs [separate covariates for each of the following drugs/drug classes: low-dose aspirin, clopidogrel, dipyridamole, vitamin K antagonist, direct oral anticoagulant, statins, thiazides and other non-loop diuretics, loop diuretics, beta blockers, calcium channel blockers, ACE-inhibitors and angiotensin II receptor blockers, nonsteroidal anti-inflammatory drugs, selective serotonin re-uptake inhibitors, and proton pump inhibitors]. All covariates were determined on index-date and based on all available data, i.e., from 10 years before baseline and up to index-date.
[3]No history of intracerebral hemorrhage at baseline (i.e., stems from comparison-cohort).
[4]First-ever intracerebral hemorrhage at baseline (i.e., stems from ICH-cohort).
[5]Adjusted for age (5-year bands), sex, and index date year (3-year bands).
[6]Adjusted for age (5-year bands), sex, index date year (3-year bands), duration of follow-up (4-year bands), comorbidity [separate covariates for each of the following: hypertension, atrial fibrillation, ischemic stroke, myocardial infarction, systemic embolism, peripheral arterial disease, venous thromboembolism, diabetes, chronic kidney failure, chronic hepatic diseases, chronic obstructive pulmonary disorder, disorders indicative of high alcohol intake, congestive heart failure, and cancer] and current use of drugs [separate covariates for each of the following drugs/drug classes: low-dose aspirin, clopidogrel, dipyridamole, vitamin K antagonist, direct oral anticoagulant, statins, thiazides and other non-loop diuretics, loop diuretics, beta blockers, calcium channel blockers, ACE-inhibitors and angiotensin II receptor blockers, nonsteroidal anti-inflammatory drugs, selective serotonin re-uptake inhibitors, and proton pump inhibitors]. All covariates were determined on index-date and based on all available data, i.e., from 10 years before baseline and up to index-date.
[7]At baseline or during follow-up.
[8]Defined as ischemic stroke or 'unspecified stroke' (only if recorded before inclusion into baseline), myocardial infarction, systemic embolism, revascularization procedures, mesenteric ischemia, venous thromboembolism, or sudden cardiac death.
[9]Only ischemic strokes recorded before baseline (i.e., before inclusion into cohorts) included.
[10]Defined as in footnote 8 but excluding ischemic stroke or 'unspecified stroke'.

© 2022 Gaist D et al. *JAMA Network Open.*

**eFigure 1.** Flow Chart of Creation of Intracerebral Hemorrhage (ICH) Cohort and Comparison Cohort Based on Data From a Previous Study

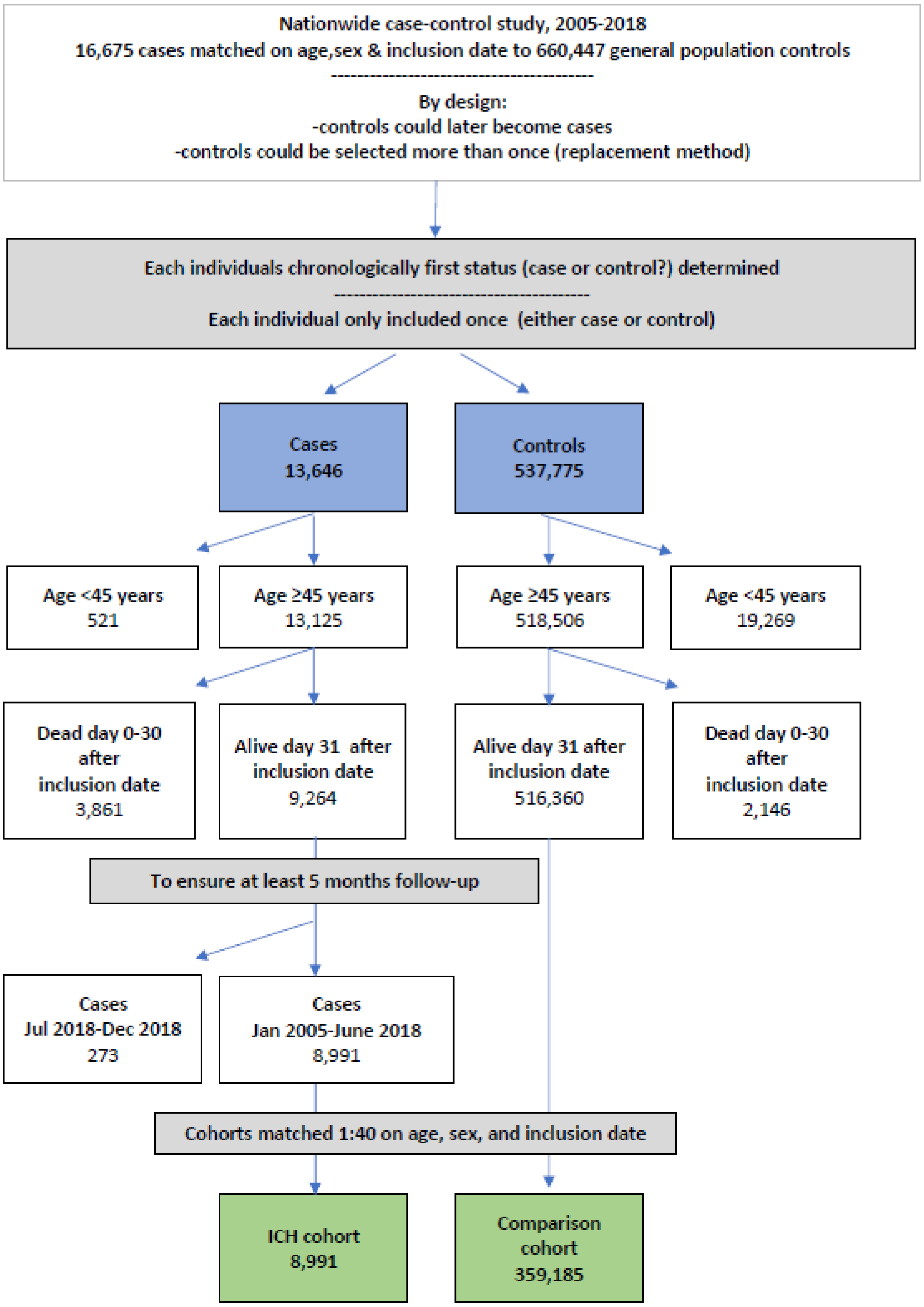


© 2022 Gaist D et al. *JAMA Network Open.*

**eFigure 2.** Cohort Design

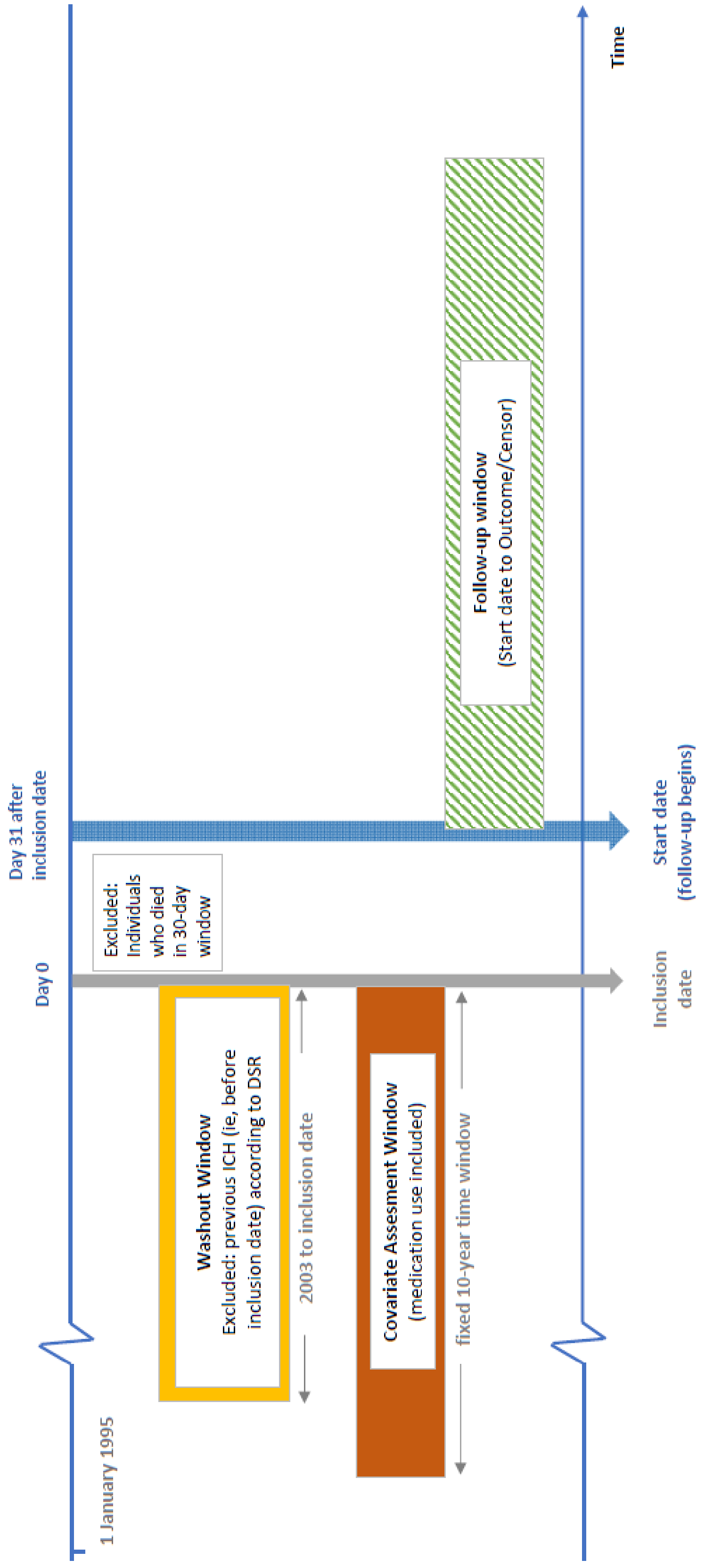


© 2022 Gaist D et al. *JAMA Network Open.*

**eFigure 3.** Nested Case-Control Design

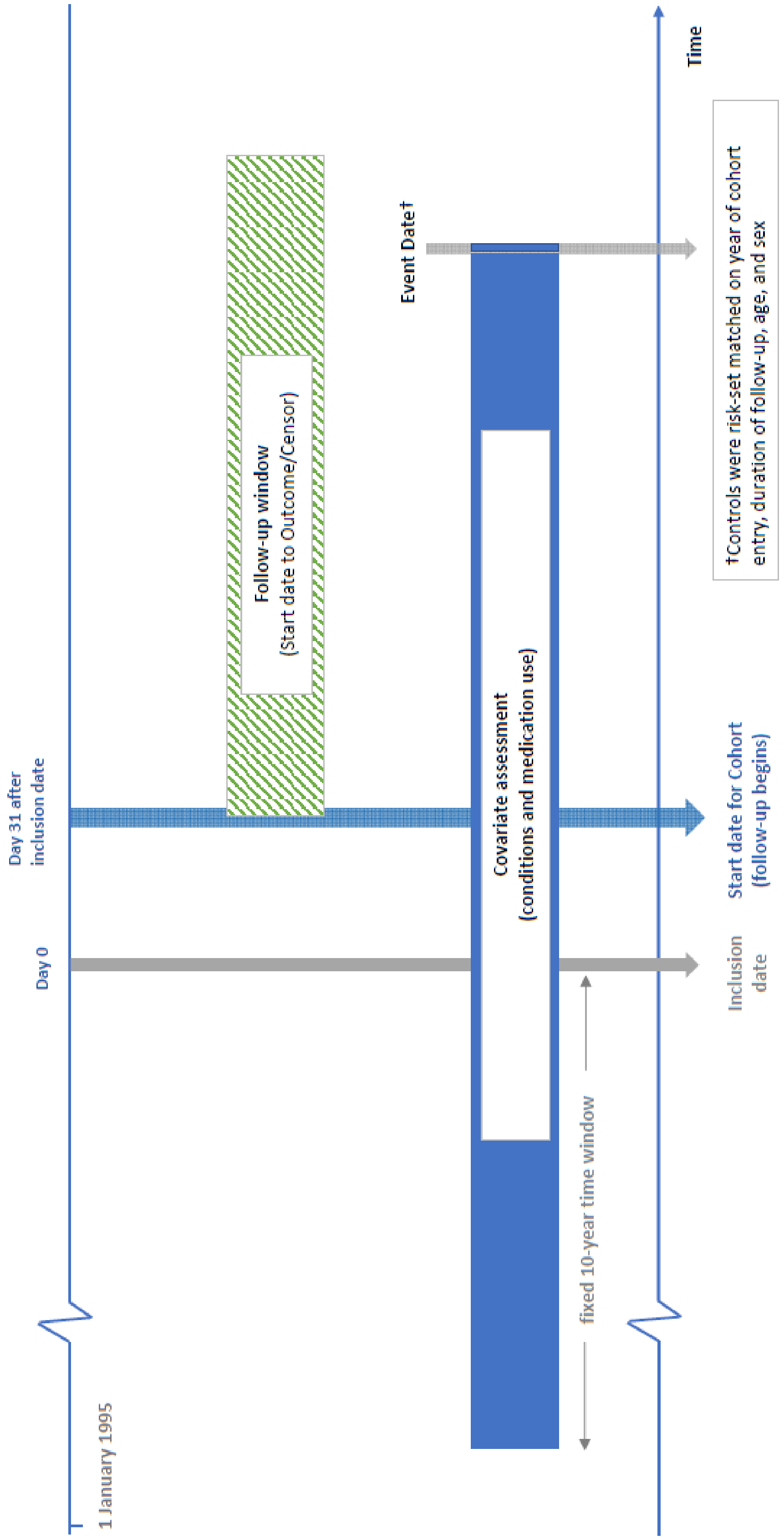


© 2022 Gaist D et al. *JAMA Network Open.*

**eFigure 4.** Cumulative Incidence of Ischemic Stroke, Intracerebral Hemorrhage, and Myocardial Infarction

**A. Cumulative Incidence**

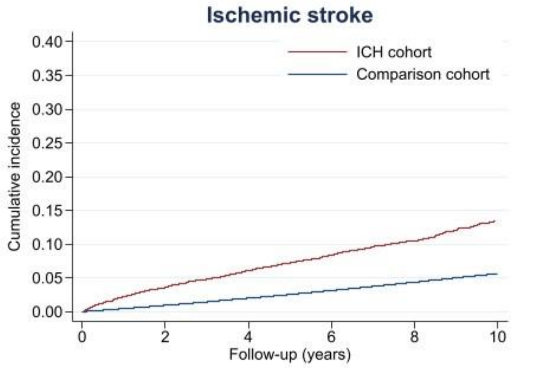


**Number at risk (number of cumulative events)**

| | | | | | | |
|---|---|---|---|---|---|---|
| Comparison cohort | 358,688 (0) | 299,764 (3,153) | 231,286 (5,986) | 172,092 (8,318) | 120,085 (10,195) | 71,858 (11,473) |
| ICH cohort | 8,991 (0) | 5,770 (266) | 3,817 (389) | 2,465 (467) | 1,519 (513) | 799 (552) |

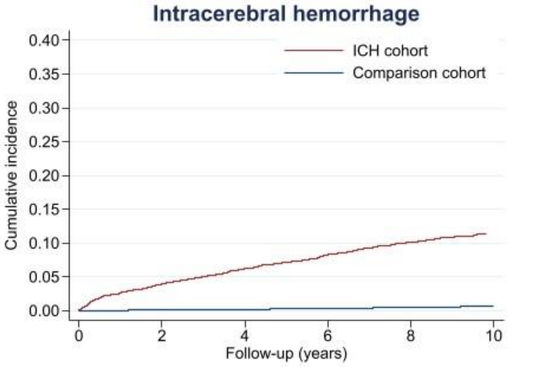


**Number at risk (number of cumulative events)**

| | | | | | | |
|---|---|---|---|---|---|---|
| Comparison cohort | 358,688 (0) | 299,764 (284) | 231,286 (560) | 172,092 (820) | 120,085 (1,047) | 71,858 (1,233) |
| ICH cohort | 8,991 (0) | 5,770 (299) | 3,817 (410) | 2,465 (477) | 1,519 (517) | 799 (534) |

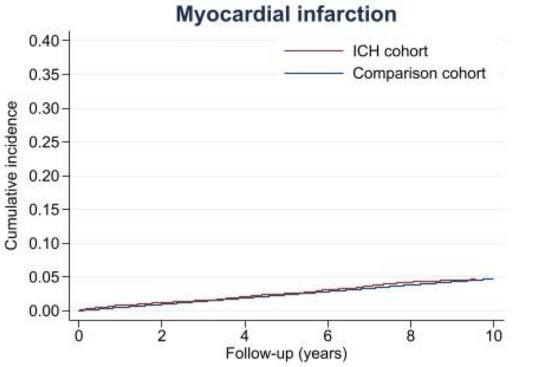


**Number at risk (number of cumulative events)**

| | | | | | | |
|---|---|---|---|---|---|---|
| Comparison cohort | 358,688 (0) | 299,764 (3,137) | 231,286 (5,678) | 172,092 (7,629) | 120,085 (9,109) | 71,858 (10,025) |
| ICH cohort | 8,991 (0) | 5,770 (89) | 3,817 (128) | 2,465 (158) | 1,519 (182) | 799 (188) |

**B. Cumulative incidence with death as competing risk**

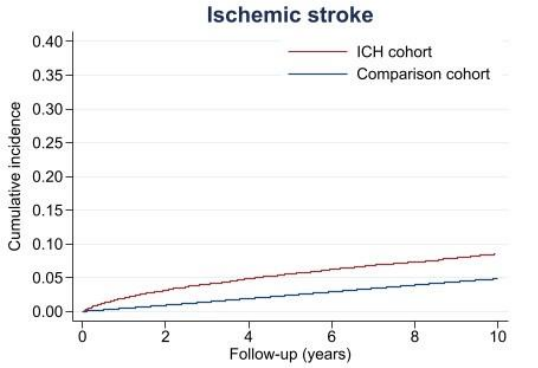


**Number at risk (number of cumulative events)**

| | | | | | | |
|---|---|---|---|---|---|---|
| Comparison cohort | 358,688 (0) | 299,764 (3,153) | 231,286 (5,986) | 172,092 (8,318) | 120,085 (10,195) | 71,858 (11,473) |
| ICH cohort | 8,991 (0) | 5,770 (266) | 3,817 (389) | 2,465 (467) | 1,519 (513) | 799 (552) |

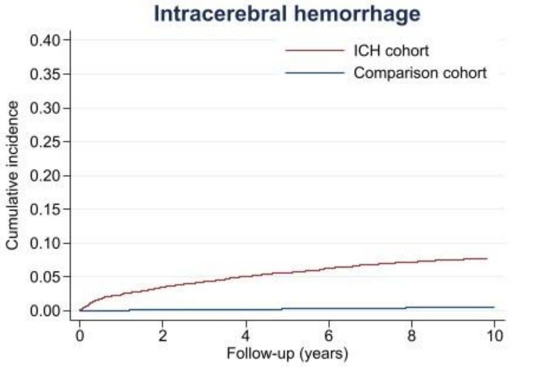


**Number at risk (number of cumulative events)**

| | | | | | | |
|---|---|---|---|---|---|---|
| Comparison cohort | 358,688 (0) | 299,764 (284) | 231,286 (560) | 172,092 (820) | 120,085 (1,047) | 71,858 (1,233) |
| ICH cohort | 8,991 (0) | 5,770 (299) | 3,817 (410) | 2,465 (477) | 1,519 (517) | 799 (534) |

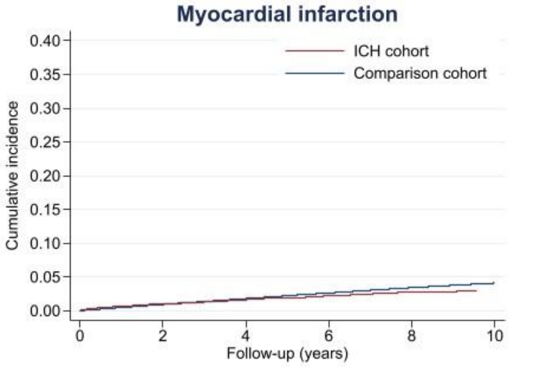


**Number at risk (number of cumulative events)**

| | | | | | | |
|---|---|---|---|---|---|---|
| Comparison cohort | 358,688 (0) | 299,764 (3,137) | 231,286 (5,678) | 172,092 (7,629) | 120,085 (9,109) | 71,858 (10,025) |
| ICH cohort | 8,991 (0) | 5,770 (89) | 3,817 (128) | 2,465 (158) | 1,519 (182) | 799 (188) |

© 2022 Gaist D et al. *JAMA Network Open.*

**eFigure 5.** Cumulative Incidence of Major Adverse Cardiovascular Event (Stroke, Myocardial Infarction, Systemic Embolism, or Vascular Death) and All-Cause Death

**A. Cumulative Incidence**

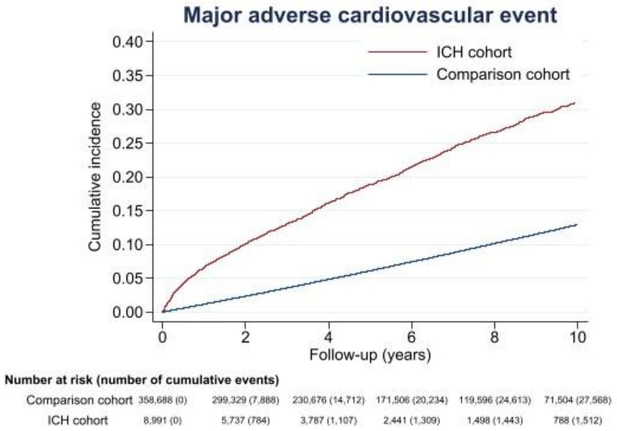


**B. Cumulative incidence with death as competing risk**

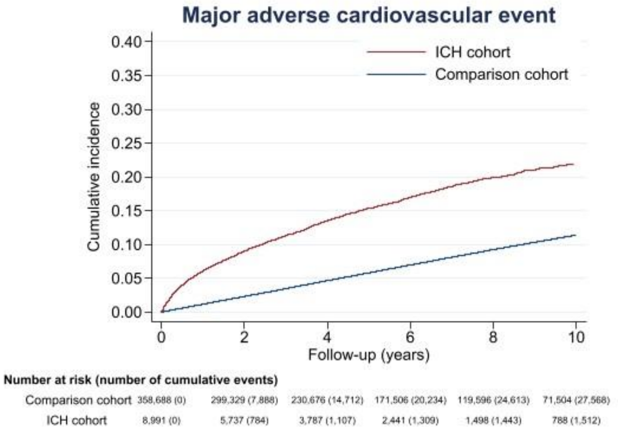


**C. All cause death**

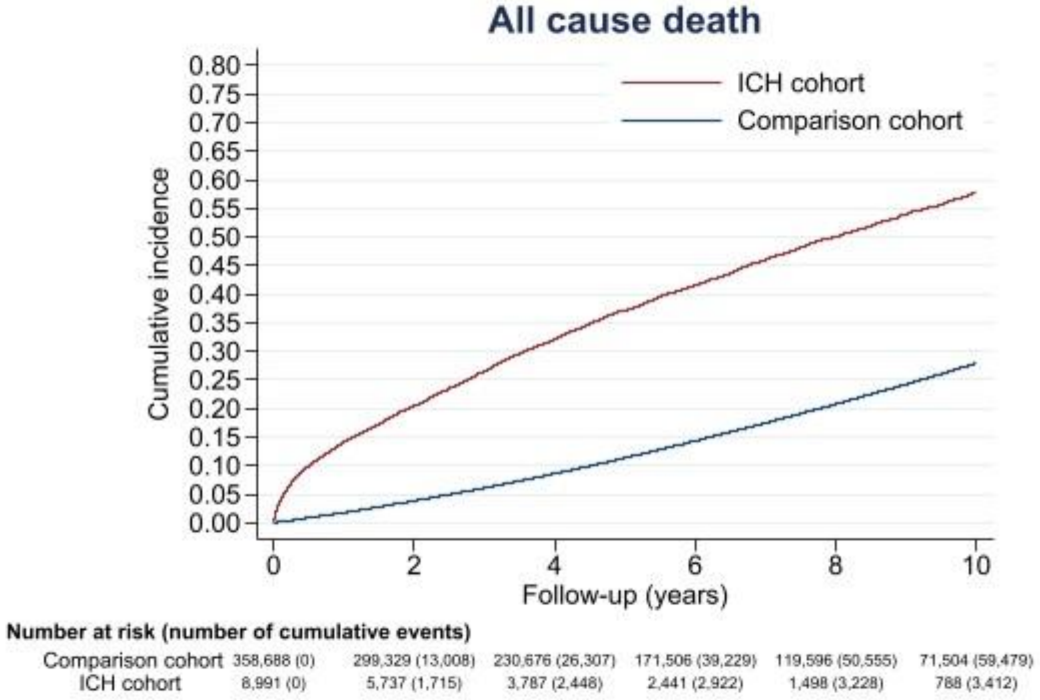


© 2022 Gaist D et al. *JAMA Network Open.*

**eFigure 6.** Cumulative Incidence of Ischemic Stroke, Intracerebral Hemorrhage, and Major Adverse Cardiovascular Event Stratified by Comorbid Atrial Fibrillation

**A. No atrial fibrillation at baseline** **B. Atrial fibrillation at baseline**

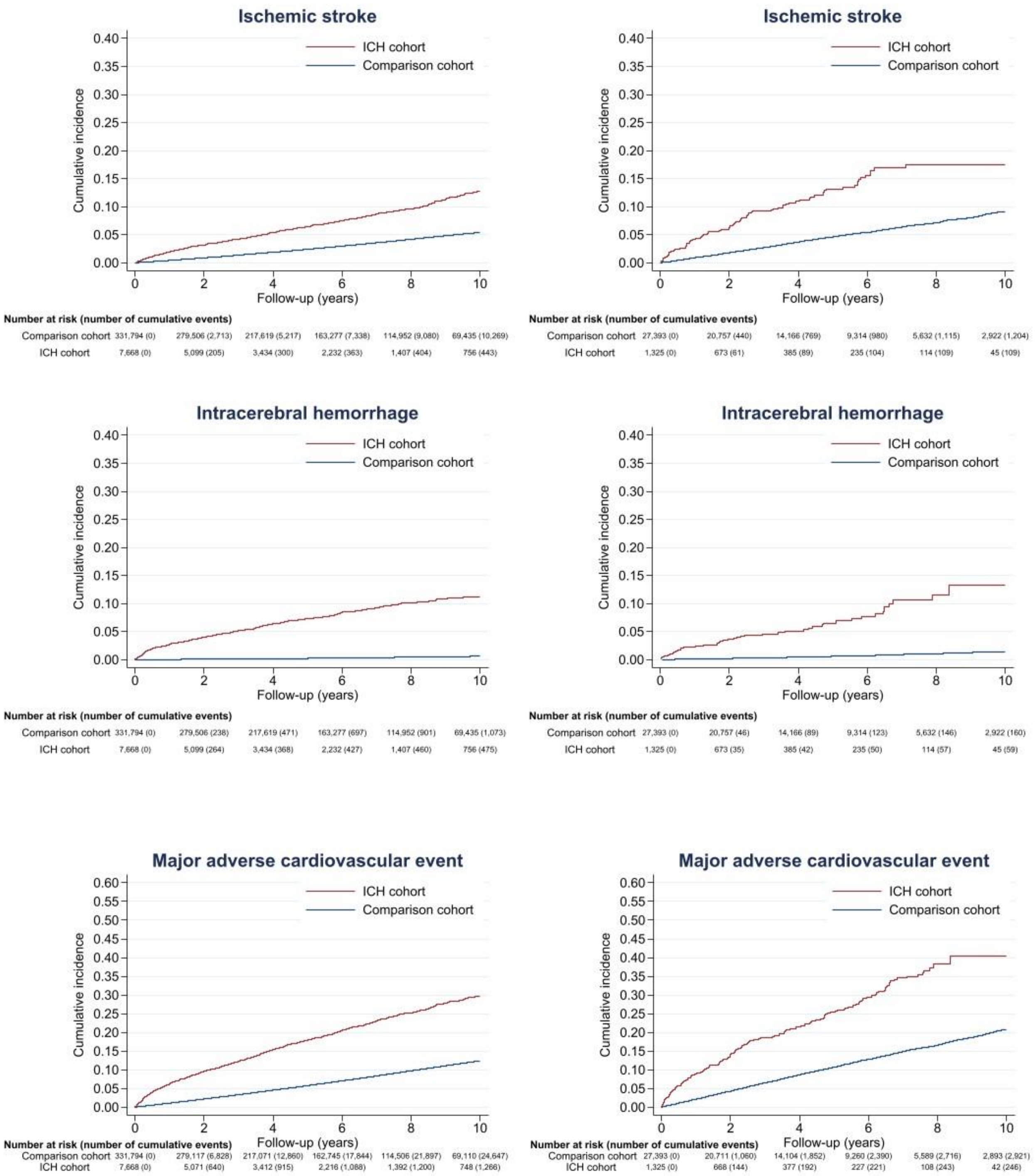


© 2022 Gaist D et al. *JAMA Network Open.*

## eReferences


1. Hald SM, Möller S, García Rodríguez LA, et al. Trends in Incidence of Intracerebral Hemorrhage and Association With Antithrombotic Drug Use in Denmark, 2005-2018. *JAMA Netw Open*. 2021;4(5):e218380. doi:10.1001/jamanetworkopen.2021.8380

2. Johnsen SP, Ingeman A, Hundborg HH, Schaarup SZ, Gyllenborg J. The Danish Stroke Registry. *Clin Epidemiol*. 2016;8:697-702.

3. Jensen MM, Hald SM, Kristensen LMB, Boe NJ, Harbo FSG, Gaist D. Validity of Simple Algorithms to Identify Recurrence of Intracerebral Hemorrhage in Two Danish Nationwide Registries. *Clin Epidemiol.* 2021;13:949-958.

4. Pottegård A, Schmidt SAJ, Wallach-Kildemoes H, Sørensen HT, Hallas J, Schmidt M. Data Resource Profile: The Danish National Prescription Registry. *Int J Epidemiol*. Published online October 27, 2016.

5. WHO Collaborating Centre for Drug Statistics Methodology. *Guidelines for ATC Classification and DDD Assignment.* WHO-2015. http://www.whocc.no/atc_ddd_index/. 2021

6. Henriksen DP, Stage TB, Hansen MR, Rasmussen L, Damkier P, Pottegård A. The potential drug-drug interaction between proton pump inhibitors and warfarin. *Pharmacoepidemiol Drug Saf.* 2015;24(12):1337-1340.


© 2022 Gaist D et al. *JAMA Network Open.*
